# Applying stability selection to consistently estimate sparse principal components in high-dimensional molecular data

**DOI:** 10.1093/bioinformatics/btv197

**Published:** 2015-04-10

**Authors:** Martin Sill, Maral Saadati, Axel Benner

**Affiliations:** Division of Biostatistics, DKFZ, 69120 Heidelberg, Germany

## Abstract

**Motivation:** Principal component analysis (PCA) is a basic tool often used in bioinformatics for visualization and dimension reduction. However, it is known that PCA may not consistently estimate the true direction of maximal variability in high-dimensional, low sample size settings, which are typical for molecular data. Assuming that the underlying signal is sparse, i.e. that only a fraction of features contribute to a principal component (PC), this estimation consistency can be retained. Most existing sparse PCA methods use L1-penalization, i.e. the *lasso*, to perform feature selection. But, the *lasso* is known to lack variable selection consistency in high dimensions and therefore a subsequent interpretation of selected features can give misleading results.

**Results:** We present S4VDPCA, a sparse PCA method that incorporates a subsampling approach, namely stability selection. S4VDPCA can consistently select the truly relevant variables contributing to a sparse PC while also consistently estimate the direction of maximal variability. The performance of the S4VDPCA is assessed in a simulation study and compared to other PCA approaches, as well as to a hypothetical oracle PCA that ‘knows’ the truly relevant features in advance and thus finds optimal, unbiased sparse PCs. S4VDPCA is computationally efficient and performs best in simulations regarding parameter estimation consistency and feature selection consistency. Furthermore, S4VDPCA is applied to a publicly available gene expression data set of medulloblastoma brain tumors. Features contributing to the first two estimated sparse PCs represent genes significantly over-represented in pathways typically deregulated between molecular subgroups of medulloblastoma.

**Availability and implementation:** Software is available at https://github.com/mwsill/s4vdpca.

**Contact:** m.sill@dkfz.de

**Supplementary information:** Supplementary data are available at *Bioinformatics* online.

## 1 Introduction

Principal component analysis (PCA) is the most popular method for dimension reduction and visualization that is widely used for the analysis of high-dimensional molecular data. In bioinformatics typical applications range from outlier detection as part of quality control (Kauffmann *et* *al.*, 2009) to exploratory data analysis for revealing new molecular subgroups (Remke *et* *al.*, 2011), as well as pathway and network analysis (Ma and Dai, 2011). Common biological data sets for such applications are continuous molecular data typically generated by high-throughput profiling techniques, e.g. gene expression, copy number variation, methylation and micro RNA expression data.

In general, PCA aims to project a high-dimensional data matrix into a lower dimensional space by seeking linear combinations of the original variables, called principal components (PCs). By construction, these PCs capture maximal variance and are orthogonal to each other. As PCs are mutually uncorrelated, PCA is a practical method to aggregate correlated variables. The resulting PCs can then be used as input variables for further analysis, e.g. principal component regression (Jolliffe, 1982). In gene expression data analysis PCs are often referred to as ’metagenes’, ’eigengenes’ or ’latent genes’. Moreover, PCs extracted from different molecular data sets can be combined to perform an integrated analysis.

Although PCA was originally developed for the multivariate normal distribution, it is not restricted to this distribution and can generally be used for exploratory data analysis and dimension reduction. However, PCA can be strongly impacted by some types of non-Gaussianity such as outliers and extreme skewness. This might be a problem for some molecular data types, but often data can be transformed to approximately achieve normality.

A major drawback of PCA is that resulting principal components are linear combinations of all variables and that the corresponding loadings vector involve only non-zero coefficients. Therefore, a practical interpretation of the loadings vectors is often complicated, especially for high-dimensional data. Furthermore, in high-dimensional, low-sample size settings (HDLSS), which are typical for molecular data sets, PCA is known to become inconsistent in estimating the leading eigenvectors of the underlying population variance covariance matrix (Jung and Marron, 2009), i.e. with increasing dimensionality and fixed sample size the estimate of the first PC does not necessarily converge towards the true direction of maximal variance.

A possible way to overcome these two drawbacks is to assume the data embodies a strong structure. This is characterized by two assumptions. First, it is assumed that the majority of variability in the data can be explained by the first few PCs and thus the data matrix can be sufficiently approximated by a matrix of lower rank. Secondly, it is assumed that only few variables contribute to the true signal of a PC. This so-called sparsity (or parsimony) assumption is supported by current knowledge about biological processes, which in most situations also involve only few genes or molecular features. In the context of PCA, we consider methods that search for PCs where only a few coefficients of the loadings vector are non-zero. So far several methods to find sparse PCA solutions have been proposed (Jolliffe *et* *al.*, 2003; Lee *et* *al.*, 2010; Shen and Huang, 2008; Witten *et* *al.*, 2009; Yang *et* *al.*, 2014; Zou *et* *al.*, 2004).

Shen *et* *al.* (2013) clearly characterized the asymptotics of sparse PCA in high-dimensional, low-sample size settings. They showed that under the assumption that the true loadings vector is sparse and given that the underlying signal is strong relative to the number of variables involved, sparse PCA methods are able to consistently estimate the direction of maximal variance. In addition, they proved that the regularized sparse PCA method (RSPCA) proposed by Shen and Huang (2008) is a consistent sparse PCA method. The focus of their work is on consistency in terms of estimating the true direction of maximal variance which corresponds to consistency in the parameter estimation of a statistical model. However, despite parameter estimation consistency, model selection consistency, i.e. selecting the variables that truly contribute to a PC, also plays an important role. Particularly in case of molecular data, selecting the correct features might be crucial for further interpretation of the PCs. For example, supposing that the selected features are subsequently analysed by downstream pathway analysis, then falsely selected irrelevant features might give misleading results.

The RSPCA algorithm applies *L*_1_-penalized ordinary least squares, also known as the *lasso* (Tibshirani, 1996), to estimate sparse loadings vectors. The *lasso* is a popular method whose model selection consistency has been widely explored in the literature (Meinshausen and Bühlmann, 2006; Zhao and Yu, 2006). The *lasso* selects variables by shrinking estimates towards zero such that small coefficients will become exactly zero. Choosing the penalization for the *lasso* usually results in a trade-off between large models with many falsely selected coefficients and small, biased models which underestimate the coefficients of truly relevant variables and thus fit the data poorly. Typically, the strength of the *L*_1_-penalization is determined by the regularization parameter *λ.* In practice, *λ* is chosen so as to optimize the goodness of fit of the model. In case of PCA methods where each PC is a rank one approximation, the goodness of fit can be measured by the Frobenius norm which corresponds to *L*_2_-norm for matrices and measures the closeness of a rank one approximation to the original data matrix. An optimal *λ* leads to sparse PC loadings vectors, where not only the coefficients of the truly relevant variables are non-zero, but also the coefficients of some irrelevant features. This is particularly meaningful for high-dimensional molecular data, where some irrelevant features are likely to be correlated with relevant features. The reason being that an optimal rank one approximation is achieved by unbiased estimates of the relevant features. To get nearly unbiased estimates penalization should not be too strong, thus increasing the chance of irrelevant features to be included in the model.

To overcome this problem of estimation bias other penalty terms have been developed. Fan and Li (2001) suggest a non-concave penalty function referred to as the smoothly clipped absolute deviation (SCAD). The adaptive *lasso* proposed by Zou (2006) uses individual weights for the penalty of each coefficient. These weights are chosen by an initial model fit, such that features that are assumed to have large effects will have smaller weights than features with small coefficients in the initial fit. Both of these penalties fulfill the oracle property, i.e. the penalized estimator is asymptotically equivalent to the oracle estimator, namely the ideal unpenalized estimator obtained when only the truly relevant variables are used for PCA.

However, even though the *lasso* does not fulfill the oracle property and can not achieve model selection consistency in high-dimensional data, it selects the truly relevant variables with high probability (Benner *et* *al.*, 2010). To utilize this property we propose to apply stability selection (Meinshausen and Bühlmann, 2010) to the *lasso* estimator involved in the RSPCA algorithm. Stability selection is a general framework to combine variable selection methods such as penalized regression models with subsampling strategies. Variable selection probabilities are estimated by applying variable selection methods to subsamples of the data, drawn without replacement, and estimating the proportion of subsamples where the variable was included in the fitted model. These selection probabilities are used to define a set of stable variables. Meinshausen and Bühlmann (2010) provide a theoretical framework for controlling Type I error rates of falsely assigning variables to the set of stable variables. Here we suggest to apply the subsampling scheme of stability selection to the *lasso* estimator involved in the RSPCA algorithm to estimate selection probabilities which are then used to identify the truly relevant variables contributing to a PC. As the *lasso* selects true variables with high probability the corresponding selection probabilities estimated with stability selection are expected to dominate those of irrelevant variables. Applying a classical forward model selection to the features ranked by these selection probabilities, sparse loadings vectors that are parameter estimation consistent as well as model selection consistent can be identified.

This manuscript is structured as follows: Section 2 describes the PCA, the RSPCA and the proposed sparse PCA method that involves stability selection. In Section 3 we describe the design and results of the simulation study that was performed to compare the different PCA methods. In Section 4 we demonstrate the practicability of the proposed sparse PCA approach by applying it to a publicly available gene expression data set of medulloblastoma brain tumors (Remke *et* *al.*, 2011). Finally, we discuss our findings and their relevance for estimating sparse PCs in high-dimensional molecular data.

## 2 Methods

### 2.1 Principal component analysis (PCA)

Suppose **X** is an *n* × *p* data matrix with entries *x_ji_* and indices j=1,…,n and i=1,…,p and rank *r*, where *p* corresponds to the number of features measured over *n* samples. Further, **X** has been mean centered such that the means of all *p* variables are zero. PCA seeks a number of K≤r linear combinations of the *p* variables that capture maximal variance:

(1) $${\tilde{u}}_{k}=X^{T}v_{k}=\sum_{i=1}^{p}v_{k,i}x_{i},$$

where ${\tilde{u}}_{k}$ is the *k*th principal component (PC), k=1,…,K and $v_{k}$ is the so-called loadings vector. $v_{k}$ has unit length and maximizes the variance of the *k*th PC. The coefficients of the loadings vector are interpreted as the contribution of each variable to the *k*th PC. Typically, the PCs are uncorrelated, i.e. the first PC points in the direction of maximal variance and the second PC shows in the direction of maximal variance orthogonal to the first PC and so on. A PCA can be performed by either an eigenvalue decomposition of the covariance matrix Σ or by singular value decomposition (SVD) of the data matrix **X**.

The SVD of **X** is:

(2) $$X=UDV^{T},$$

where **U** is a *n* × *r* orthogonal matrix and the column vectors $u_{k}$ are the PCs scaled to unit length. **V** is a *p* × *r* orthogonal matrix with columns $v_{k}$, which represent the loadings vectors and are equal to the eigenvectors of the sample covariance matrix $\hat{\Sigma }$. **D** is a diagonal matrix and the diagonal entries $d_{1},\ldots ,d_{r}$ are the singular values, where $d_{k}u_{k}={\tilde{u}}_{k}$ is the *k*th PC with variance $d_{k}^{2}$. Typically, we are interested in a low-rank approximation of **X**, i.e. the first few PCs that explain most of the variance. It is known that the SVD gives the closest rank one approximation of **X** with respect to the Frobenius norm (Eckart and Young, 1936):

(3) $$(d,u,v)={\text{arg }\underset{d,u,v}{\text{min}}}_{}\|{X-duv^{T}\|}_{F}^{2},$$

where ${\left||\cdot |\right|}_{F}^{2}$ indicates the squared Frobenius norm, which is the sum of squared elements of the matrix.

### 2.2 Regularized sparse principal component analysis (RSPCA)

Shen and Huang (2008) and later Lee *et* *al.* (2010) showed that, with **u** fixed, the minimization in Equation (3) can be formulated as a least squares regression. For fixed **u**, the least squares coefficient vector of regressing the columns of **X** on **u** is $\tilde{v}=dv$. The ordinary least squares estimator (OLS) for $\tilde{v}$ is $\hat{\tilde{v}}=Xu$. Without loss of generality, holding **v** fixed the OLS for $\tilde{u}$ is $\hat{\tilde{u}}=X^{T}v$. With this connection to least squares regression it is straightforward to use penalization terms to impose sparsity on $\tilde{v}$.

(4) $$(u,\hat{\tilde{v}})={\text{arg }\underset{u,\tilde{v}}{\text{min}}}_{}\|{X-u{\hat{\tilde{v}}}^{T}\|}_{F}^{2}+\lambda P(\tilde{v}),$$

where $P(\tilde{v})$ is a penalization term that induces sparsity on $\tilde{v}$ and *λ* is a tuning parameter that determines the strength of the penalization. The RSPCA algorithm uses the *lasso* penalty $P(\tilde{v})=|\tilde{v}|$, however other sparsity inducing penalization terms such as the adaptive *lasso* (Zou, 2006) and the SCAD-penalty (Fan and Li, 2001) are conceivable. With the *lasso* penalization term in Equation (4) a soft-thresholding estimator (Tibshirani, 1996) can be derived to estimate the elements of $\hat{\tilde{v}}$:

(5) $${\hat{\tilde{v}}}_{i}=\text{sign}\left\{{\left(Xu\right)}_{i}\right\}{\left(|{\left(Xu\right)}_{i}|-\lambda \right)}_{+}.$$

Using adaptive *lasso* weights, the soft-thresholding estimator is given by:

(6) $${\hat{\tilde{v}}}_{i}=\text{sign}\left\{{\left(Xu\right)}_{i}\right\}{\left(|{\left(Xu\right)}_{i}|-{\hat{w}}_{i}\lambda \right)}_{+}.$$

where the ${\hat{w}}_{i}$’s are weights chosen by an initial model fit $\hat{w}=1/Xu^{\gamma }$. Here *γ* determines the strength of the weighting, typical values are in the range 0<γ≤2. Using the SCAD-penalty, the estimator is given by:

(7) $${\hat{\tilde{v}}}_{i}=\{\begin{array}{ll} \text{sign}\left\{{\left(Xu\right)}_{i}\right\}(|{\left(Xu\right)}_{i}|-\lambda ) & \text{ if }|{\left(Xu\right)}_{i}|\leq \lambda \\ \text{sign}\left\{{\left(Xu\right)}_{i}\right\}(|{\left(Xu\right)}_{i}|-\frac{a\lambda -\left\{{\left(Xu\right)}_{i}\right\}}{a-1}) & \text{ if }\lambda <|{\left(Xu\right)}_{i}|\leq a\lambda \\ Xu_{i} & \text{ if }|{\left(Xu\right)}_{i}|>a\lambda \end{array}$$

Here *a* > 2 is a tuning parameter. Fan and Li (2001) showed that the SCAD prediction is not sensitive to selection of *a* and suggest to use *a* = 3.7. The SCAD-penalty function corresponds to a quadratic spline function with knots at *λ* and aλ, which leaves large values of the vector $\hat{\tilde{v}}$ not excessively penalized.

Lee *et* *al.* (2010) proposed an algorithm that solves the minimization problem in Equation (4). Using the *lasso* estimator in Equation (5) the algorithm alternates between the following two steps until convergence:

1. ${\hat{\tilde{v}}}_{i}=\text{sign}\left\{{\left(Xu\right)}_{i}\right\}{\left(|{\left(Xu\right)}_{i}|-\lambda \right)}_{+}$ $v=\hat{\tilde{v}}/\|\hat{\tilde{v}}\|$
2. $\hat{\tilde{u}}=X^{T}v$ $u=\hat{\tilde{u}}/\|\hat{\tilde{u}}\|$

To choose an optimal penalization parameter *λ*, Lee *et* *al.* (2010) proposed to use the Bayesian Information Criterion (BIC). The BIC is a model selection criterion related to Bayesian variable selection that assesses the quality of a model by the goodness of fit while penalizing for the complexity of the model, i.e. the number of parameters in the model.

(8) $$BIC(\lambda )=\frac{\|{X-duv^{T}\|}_{F}^{2}}{np{\hat{\sigma }}^{2}}+\hat{df}(\lambda )\frac{log(np)}{np},$$

where $\hat{d}f(\lambda )$ is the degree of sparsity of the loadings vector **v** with penalty parameter *λ*, and ${\hat{\sigma }}^{2}$ is the OLS estimate of the error variance of the model. Subsequent PCs are fitted by subtracting the rank one approximation corresponding to the estimated sparse PC from the data matrix and applying the algorithm to the residual matrix.

### 2.3 Sparse PCA by sparse SVD using stability selection (S4VDPCA)

In contrast to the approach described so far, we propose to identify the variables that truly contribute to the leading eigenvector by applying a subsampling technique motivated by stability selection (Meinshausen and Bühlmann, 2010). By applying the corresponding variable selection method to subsamples drawn without replacement, selection probabilities for each variable can be estimated as the proportion of subsamples where the variable is included in the fitted model. The selection probability of each variable along the regularization path, e.g. along the range of possible penalization parameters, is called the stability path. Here we propose to estimate the selection probabilities of the variables that contribute to sparse PCs by applying this resampling scheme to the *lasso* estimator as defined in Equation (5).

In addition, we adopt the idea of the ‘randomized *lasso*’ also described by Meinshausen and Bühlmann (2010). In each resampling iteration and for each of the *p* components of $\hat{\tilde{v}}$ a randomized reweighing of the penalization parameter *λ* is performed. In each iteration weights $w_{1},\ldots ,w_{p}$ are sampled from a uniform distribution, i.e. $w_{i}∼U(\kappa ,1)$. Given these weights the ’randomized *lasso*’ estimator is:

(9) $${\hat{\tilde{v}}}_{i}=\text{sign}\left\{{\left(Xu\right)}_{i}\right\}{\left(|{\left(Xu\right)}_{i}|-\frac{\lambda }{w_{i}}\right)}_{+}.$$

In this context, the so called weakness parameter κ∈(0,1] describes the amount of additional randomization and the ‘randomized *lasso*’ changes the penalization parameter *λ* to a randomly chosen value in the range of [λ,λ/κ]. Meinshausen and Bühlmann (2010) showed that this additional randomization achieves model selection consistency even in situations where the necessary conditions for consistency of the *lasso* are violated. The ‘randomized *lasso*’ decorrelates variables and therefore addresses the model selection inconsistency problem of standard *lasso* in the presence of correlations between relevant and irrelevant variables. According to Meinshausen and Bühlmann (2010) a low value of *κ* lowers the probability of irrelevant variables to be selected. They propose to choose *κ* in the range (0.2, 0.8) in applications.

Due to computational complexity we do not calculate the whole stability path but follow the idea of point-wise control described by Meinshausen and Bühlmann (2010) and choose a single *λ* at which selection probabilities are estimated. This *λ* should not penalize too strong so that in each iteration of the stability selection the true non-zero coefficients are selected with high probability. To find such a *λ*, we estimate the selection probabilities for several possible penalization parameter and choose the lambda that leads to minimal number of ties in the selection probabilities.

Ranking the variables according to their estimated selection probability a forward selection procedure is applied: starting with the variable with highest selection probability, we subsequently add variables and calculate sparse PCA solutions by applying regular SVD to the reduced matrix involving only the variables with highest selection probability. The remaining coefficients of $\hat{v}$ that correspond to variables with lower selection probability, are set to zero. The final sparse PCA solution can be selected by applying a model selection criterion. It is known that model selection criteria like the BIC used in the RSPCA may select more variables than necessary when the number of variables is larger than the number of observations. Instead, a generalized information criterion (GIC) according to Kim *et* *al.* (2012) is applied:

(10) $$\text{GIC}(\lambda )=\frac{\|{X-duv^{T}\|}_{F}^{2}}{np{\hat{\sigma }}^{2}}+\hat{d}f(\lambda )\frac{\text{log}(\text{log}(np))\text{log}(p)}{np},$$

Further PCs can be fitted by subtracting the rank one approximation, i.e. the estimated sparse PC, from the data matrix and applying the algorithm to the resulting residual matrix.

## 3 Simulation study

### 3.1 Study design

In a simulation study the proposed S4VDPCA method is compared to conventional PCA, the RSPCA with *lasso* penalty, with adaptive *lasso* penalty and with SCAD penalty. Furthermore, these methods are also compared to that of an oracle PCA, i.e. a PCA that ‘knows’ the coefficients of the true PC solution. In order to guarantee comparability between the S4VDPCA and the RSPCA with different penalty functions, the BIC used in Lee *et* *al.* (2010) to choose an optimal penalization parameter within the RSPCA algorithm is replaced by the GIC of Equation (10). Moreover, the tuning parameter *γ* used in the adaptive *lasso* in Equation (6) was set to *γ* = 1 for all simulations. In the same way, parameter *a* of the SCAD-penalty in Equation (7) was set to *a* = 3.7. The number of iterations for the stability selection was set to 500 and the weakness parameter was set to κ=0.2.

To simulate data the underlying true population covariance matrix Σ was generated according to the single-covariance spike model described by Amini and Wainwright (2008):

(11) $$\Sigma =(d-1)vv^{T}+I_{p}.$$

Here $d=p^{\alpha }$ is the simulated, leading eigenvalue and *α* is the spike index 0≤α, i.e. the dominance of the eigenvalue. **v** is the corresponding sparse eigenvector, the true loadings vector, of length *p*, where $\left\lfloor p^{\beta }\right\rfloor$ coefficients of **v** are non-zero with value $1/\sqrt{\left\lfloor p^{\beta }\right\rfloor }$, such that ||v||=1. *β* is the sparsity index that measures the sparsity of **v** and is in the range of 0≤β≤1. For the simulation study all combinations of *α* and *β* from 0.1 to 1 with step size 0.05 were investigated. At each point of this considered parameter space, Σ was generated for *p* = 1000 features using the formula of the single-covariance spike model in Equation (11). Given Σ, 100 data matrices with sample size *n* = 50 were generated by sampling from a multivariate normal distribution X∼N(μ,Σ), where μ is a zero vector of length *p.* To estimate **v**, S4VDPCA, RSPCA, conventional PCA and the oracle PCA were applied to these matrices. Oracle PCA estimates are calculated by applying regular SVD to a reduced matrix that involves only variables that are known to have non-zero coefficients in **v**. The remaining coefficients of $\hat{v}$ that correspond to the zero entries in **v** are set to zero.

To evaluate the results regarding parameter estimation consistency, the angle between the true loadings vector, the leading eigenvector of Σ, and the estimates are calculated,

(12) $$A(\hat{v},v)\equiv \arccos\left|\langle \hat{v},v\rangle \right|.$$

Here *A* denotes the angle and 〈•,•〉 is the inner product.

Following Shen *et* *al.* (2013) an estimator of **v** is considered *consistent* as long as $A(\hat{v},v)\overset{p}{\to }0$. Moreover, an estimator is considered *marginally inconsistent* if $A(\hat{v},v)\overset{p}{\to }(0,\frac{\pi }{2})$ and *strongly inconsistent* if $A(\hat{v},v)\overset{p}{\to }\frac{\pi }{2}$. In theory, sparse PCA methods are able to consistently estimate **v** in high-dimensional, low-sample size data as long as 0≤β<α≤1, but are marginally inconsistent if β=α and strongly inconsistent if β>α. For situations in which the signal of the leading eigenvector is relatively strong, i.e. α≥1, even conventional PCA is expected to give consistent estimates (Jung and Marron, 2009). In addition, to assess whether the different PCA methods select the true non-zero coefficients in **v** the false discovery rates (FDR) were calculated.

### 3.2 Results

**Fig. 1.**
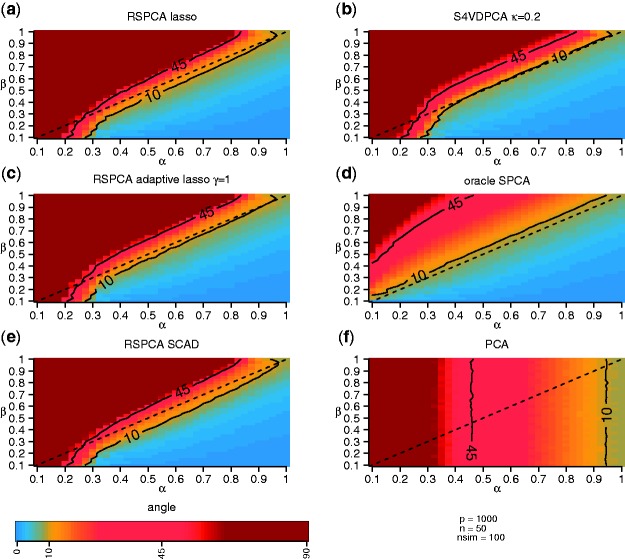
Angles between estimated and true leading eigenvector for (**a**) RSPCA with *lasso* penalty, (**b**) S4VDPCA, (**c**) RSPCA with adaptive *lasso* penalty, (**d**) *oracle* SPCA, (**e**) RSPCA with SCAD penalty and (**f**) conventional PCA. The colors correspond to the median angle calculated over 100 simulation runs. Angles with 10 and 45 degrees of deviation are indicated by contour lines. The sparsity index *β* and the spike index *α* define the sparsity, e.g. the number of truly non-zero coefficients, and the dominance of the signal, e.g. the eigenvalue, of the simulated first PC. Further, *p* and *n* denote the number of features and samples of the simulated data sets and *nsim* denotes the number of simulated data sets

The results of the simulation study comparing the S4VDPCA to RSPCA using different penalization functions, conventional PCA and oracle PCA are shown in Figures 1

and 2

**Fig. 2.**
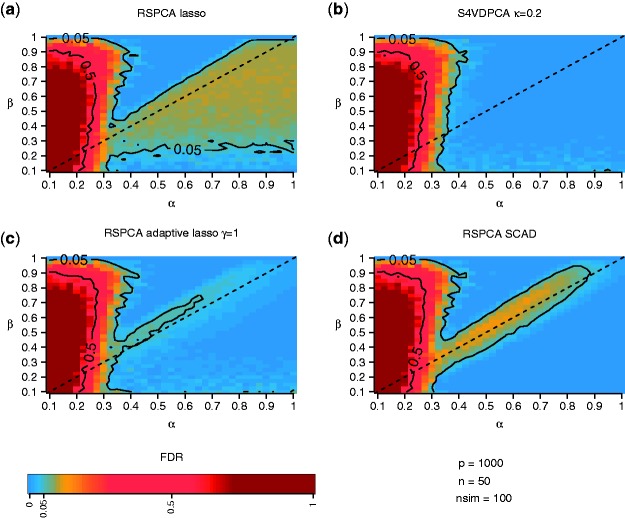
Median FDR for (**a**) RSPCA with *lasso* penalty, (**b**) S4VDPCA, (**c**) RSPCA with adaptive *lasso* penalty and (**d**) RSPCA with SCAD penalty. FDRs of 0.05 and 0.5 are indicated by contour lines. The sparsity index *β* and the spike index *α* define the sparsity, e.g. the number of truely non-zero coefficients, and the dominance of the signal, e.g. the eigenvalue, of the simulated first PC. Further, *p* and *n* denote the number of features and samples of the simulated data sets and *nsim* denotes the number of simulated data sets

. Figure 1 displays the median angles between the estimated and the true loadings vectors on a heat color scale. Simulation scenario were defined by all possible combinations of the spike index *α* and the sparsity index *β*, the median was calculated over 100 simulation runs. Furthermore, the 10 and 45 median angles are indicated by contour lines. In addition, true positive rates (TPR) and simulation results for other tuning parameters are shown in the Supplementary Material., i.e. RSPCA using the adaptive lasso with γ=0.5 and *γ* = 2 and S4VDPCA with weakness parameter κ=0.5 and κ=0.8.

As already shown in theory by Jung and Marron (2009), conventional PCA is strongly inconsistent as long as the strength of the underlying signal is weak, i.e. for spike indices α≲0.45. With increasing *α* the PCA estimates get closer to the true eigenvector, thereby achieving marginal consistency for α≳0.45 (as indicated by the 45 degree contour line) and consistency for α≳1. The behaviour of the consistency is independent of the sparsity of the underlying signal, e.g. the sparsity index *β* (Fig. 1f).

However, if the underlying signal is sparse and β<α, all considered sparse PCA methods can consistently estimate the first loadings vector and become marginally consistent for β=α (Fig. 1a–e). The oracle PCA, which ‘knows’ the true non-zero coefficients, gives unbiased estimates of the non-zero coefficients and therefore the best possible sparse solutions that are closest to the underlying first eigenvector of the population covariance matrix. The sparse loadings vectors estimated by the S4VDPCA are in all situations slightly closer to the unbiased, oracle estimates than RSPCA estimates using any considered penalty function. For β>0.2 the 10 degree contour line of the S4VDPCA is always closest to the marginal consistency boundary (β=α). Furthermore, the 45 degree contour line lies in most situations further to the left hand side of the marginal consistency boundary. If the true eigenvector is very sparse and the signal is weak, i.e. β<0.3 and α<0.3, both the RSPCA and the S4VDPCA become marginally inconsistent in estimating the true eigenvector, even if α>β.

Figure 2 displays the median FDR for the different RSPCA methods (Fig. 2a, c and d) and the S4VDPCA (Fig. 2b). The median FDR was calculated over 100 simulation runs and for all combinations of *α* and *β*, as described in the simulation design above. The FDR is shown on a heat color scale and median levels of 0.05 and 0.5 are indicated by contour lines. For relatively weak signals, starting at α=0.4, the RSPCA methods and S4VDPCA tend to falsely select coefficients resulting in FDRs around 0.05, especially in situations where the true signal is less sparse β>0.5. When α≤0.25 and β<0.9 the FDR increases dramatically to 0.5. In situations where sparse PCA methods are expected to consistently estimate the direction of the true eigenvector, i.e. α>β and β>0.2, the FDR for the RSPCA with the *lasso* is around 0.05 (Fig. 2a). This expected behaviour reflects the known variables selection inconsistency of the *lasso* in high dimensions (Meinshausen and Bühlmann, 2006; Zhao and Yu, 2006). In these simulation settings the unbiased coefficients are relatively large (α>β), so that the penalization chosen by the GIC is not sufficient to screen out irrelevant variables that are correlated with truly relevant variables. Both the adaptive *lasso* and SCAD penalty are known to possess the oracle property and are thus expected to select only truly relevant variables and achieve approximately unbiased estimates. Nevertheless, the simulation results show that both penalties tend to select additional irrelevant variables in simulation settings where the signal intensity *α* and sparsity *β* are nearly equal (depicted by the orange tail along the diagonal in Fig. 2a, c and d), i.e. around the marginal consistency boundary (β=α). This behaviour is more pronounced for the SCAD penalty compared to the adaptive lasso. In contrast, in almost all simulation settings where α>β the S4VDPCA identifies the true non-zero coefficients without adding any irrelevant features. Therefore, we can conclude, that particularly in the challenging settings where β≈α, the selection probabilities estimated by the S4VDPCA can successfully be used to filter the truly relevant features withouth selecting as many false positves as the RSPCA methods.

## 4 Application

**Fig. 3.**
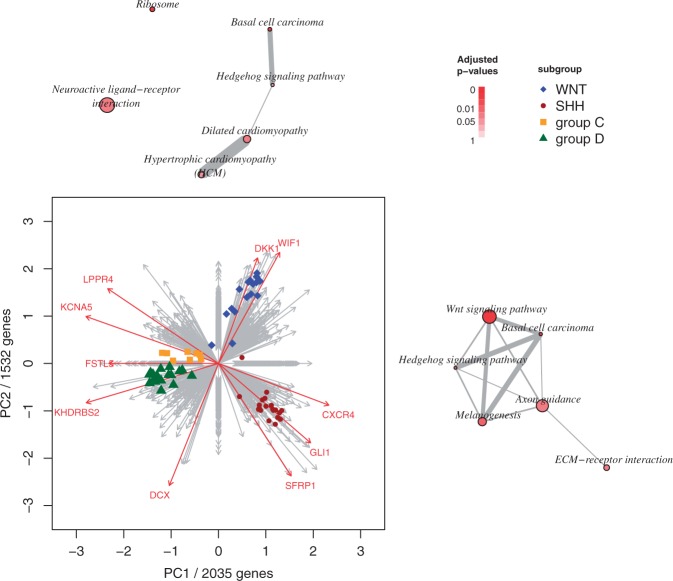
Biplot representation of the first two sparse PCs. The biplot displays the projection of the samples into the two dimensional space spanned by the first two sparse PCs. The arrows show the contribution of the selected genes to the two sparse PCs, i.e. the covariance structure of the selected genes. Each arrow represents a gene and the length of the arrow reflects the size of the corresponding coefficient in the two loadings vectors. Relevant oncogenes are highlighted in red. The nodes of the two graphs above and on the right side of the biplot represent pathways significantly overrepresented by the genes selected in the first and second PC, respectively

To demonstrate practicability to find sparse and interpretable PCs in high-dimensional molecular data sets, the proposed S4VDPCA method was applied to a gene expression data set of medulloblastoma brain tumors (Remke *et* *al.*, 2011). Medulloblastoma is the most common malignant pediatric brain tumor and comprises four distinct molecular variants. These subgroups are known as WNT, SHH, group C and group D. WNT tumours show activated *Wnt signaling pathway* and carry a favourable prognosis. SHH medulloblastoma show *Hedgehog signaling pathway* activation and are known to have an intermediate to good prognosis. While both WNT and SSH variants are molecularly already well characterized, the genetic programs driving the pathogenesis of group C and group D medulloblastoma remain largely unknown. Here we applied the proposed S4VDPCA method to gene expression data of 8 group C, 20 group D, 20 SHH and 16 WNT tumors. Gene expression has been measured by the 4x44K Agilent Whole Human Genome Oligo Microarray. After normalization and quality control the data set comprised gene expression values of 18406 annotated genes. The data set is publicly available at the National Center for Biotechnology Information (NCBI) Gene Expression Omnibus (GEO) database, Accession No. GSE28245. The first two sparse PCs have been extracted by applying the S4VDPCA and the results are visualized as biplot representation in Figure 3

. The loadings vector of the first sparse PC comprises 2035 non-zero coefficients and the second PC involves 1532 non-zero coefficients. The biplot displays the projection of the tumor samples onto the two sparse PCs while also visualizing the covariance structure of the selected genes within this rank two approximation by grey arrows. Each arrow represents a gene and the length of the arrow reflects the size of the corresponding coefficient in the two loadings vectors. Arrows that point in similar directions represent positive correlated genes. Arrows parallel to a PC axis are genes with a non zero loadings coefficient only in one of the two loadings vectors of the two PCs.

The four molecular subgroups can clearly be separated by projecting the samples in the space spanned by the first two sparse PCs. While most WNT and SHH medulloblastomas form clusters far away from samples of other subgroups, group D and group C tumors are closer to each other. A set of 2035 genes is still too large for a reasonable interpretation, but the most dominating genes, i.e. those showing the highest absolute coefficients can be highlighted.

In Figure 3, 15 prominent oncogenes with a high absolute coefficient have been highlighted, including SFRP1 and its transcription factor GLI1. Both arrows point away from the WNT samples into the direction of SHH medulloblastoma. This means that the SFRP1 expression is up-regulated in SHH tumors and down-regulated in WNT sample and matches the current knowledge that SFRP1 is a tumor suppressor gene responsible for Hedgehog signaling mediated regulation of the WNT signaling pathway. Moreover, the arrows of DKK1 and WIF1, which are known target genes of the WNT signaling pathway, both point in direction of the WNT medulloblastoma. FSTL5, a known marker for poor prognosis in non-WNT/non-SHH medulloblastoma (Remke *et* *al.*, 2011), points into a direction of group C and group D tumors. Since the loadings coefficient of FSTL5 is zero in the second PC the arrow for FSTL5 is parallel to the first PC.

However, individual interpretation of all non-zero coefficients is still too complex. An alternative way to try to understand the importance of the genes selected by sparse PCA methods is to perform a pathway analysis. Here we performed hypergeometric testing of the genes selected in the first and second PC to evaluate whether these genes are overrepresented in KEGG pathways (Kyoto Encyclopedia of Genes and Genomes; Kanehisa and Goto, 2000). To perform this analysis the R/Bioconductor package *HTSanalyzeR* was used (Wang *et* *al.*, 2011). The top six pathways most significantly overrepresented by genes selected in the first and second PC are shown as graphs above and to the right of the biplot. Each node or circle of the graph represents a pathway and the size of each node is proportional to the number of genes assigned to that pathway. Pathways are connected by edges and the width of each edge is proportional to the number of genes shared by two pathways. The white-red coloring of the nodes corresponds to FDR adjusted p-values that are also shown in the Supplementary Material. Among the top six pathways overrepresented by genes selected in the first sparse PC are the *Wnt signaling pathway* and *Neuroactive ligand-receptor interaction.* Even though these pathways include a wide range of genes, both pathways are expected to be deregulated in medulloblastoma. Interestingly, genes selected in the second, sparse PC are also significantly overrepresented in the *Wnt signaling pathway* and the *Hedgehog signaling pathway.* This result directly reflects the known interaction between these two pathways and is in agreement with the biplot where the largest distances between WNT and SHH samples are along the axis of the second PC.

Similar results calculated by applying conventional PCA, the RSPCA with *lasso*, RSPCA adaptive *lasso* and the RSPCA with SCAD penalty are shown in the Supplementary Material.

## 5 Discussion and conclusion

Here we have presented a simple and computationally efficient two-step approach to estimate consistent sparse PCA solutions in high-dimensional, low sample-size situations. In a first step features are ranked by applying a subsampling scheme motivated by stability selection. In the second step a sparse PC is estimated by simple forward selection. While existing sparse PCA methods like the RSPCA focus on finding sparse PCs that are consistent in estimating the true direction of maximal variation, the proposed S4VDPCA also takes model selection consistency into account. Model selection consistency, i.e. selecting truly relevant variables is important for a correct interpretation and further downstream analysis, e.g. performing a subsequent pathway analysis.

The stability selection applied within the S4VDPCA can be understood as an ensemble method such as bootstrap aggregation (Bagging; Breiman, 1996a). Bagging is a popular method to estimate models with improved prediction performance by reducing the variance of a single weak prediction model by aggregating the predictions of several weak models that were fitted on bootstrap samples. Similarly, by counting the number of times a variable is selected in each of the sampled subsets, the stability selection combines the information of a collection of *lasso* models. Each of these *lasso* models is weak in model selection as it suffers from the model selection inconsistency of the *lasso.* Therefore, the selected features vary, e.g. are unstable, when compared over all models in the collection. However, ranking features by estimated selection probabilities, i.e. the proportion of subsamples where the variable is included in a fitted model, allows the S4VDPCA to identify the truly relevant molecular features as variables with high selection probabilities. The additional randomization of the ‘randomized *lasso*’ approach further decorrelates variables and leads to larger differences between the selection probabilities of correlated irrelevant and relevant variables. In the same spirit other ensemble methods like the popular Random Forests algorithm (Breiman, 2001) decorrelate variables by limiting the number of variables that are allowed to be selected for each subsample.

Surprisingly, in some simulation scenarios, i.e. when the spike index *α* and sparsity index *β* are close to each other, the S4VDPCA even outperformed the RSPCA with SCAD and the adaptive *lasso* penalty. Both of these penalization functions are explicity designed to overcome the model selection inconsistency of the *lasso* and were expected to consistently select only truly relevant variables.

Applying subsampling or bootstrapping to estimate selection frequencies that are then used to rank variables for classical forward model selection can be seen as a computational shortcut to a stable ‘best’ subset selection. The best subset selection is a combinatorial procedure which evaluates all subsets by minimizing some selection criterion like the BIC. Conventional best subset selection is not feasible for high-dimensional data and is known to suffer from instability in variable selection (Breiman, 1996b). The two-step approach of the S4VDPCA addresses the instability and the computational complexity by applying stability selection to rank variables. Moreover, this two-step procedure is a rather general idea that could be applied to all kinds of statistical prediction problems to find parameter consistent and model selection consistent estimates in high dimensions.

The computational bottleneck for both the RSPCA and the S4VDPCA algorithm is the optimization of the information criterion, here the BIC or GIC. These information criteria are step functions of *p* that involve computationally costly matrix multiplications to calculate the goodness of fit of the sparse rank one approximation. To reduce the computation time we have implemented a parallelized search algorithm that can be used for both sparse PCA methods. However, by design the RSPCA optimizes the information criterion in each iteration of the algorithm while the S4VDPCA performs the stability selection once and requires only a single information criteria optimization step to select the final model via forward selection. In addition, the stability selection is an embarrassingly parallel computation problem that can easily be solved using parallel implementation.

## Supplementary Material

Supplementary Data

## References

[btv197-B1] Amini A.A. Wainwright M.J. (2008) High-dimensional analysis of semidefinite relaxations for sparse principal components. In: Information Theory, 2008. ISIT 2008. IEEE International Symposium*,* pp. 2454–2458. IEEE.

[btv197-B2] Benner A. (2010) High-dimensional Cox models: the choice of penalty as part of the model building process. Biometr. J.*,* 52, 50–69. 10.1002/bimj.200900064 20166132

[btv197-B3] Breiman L. (1996a) Bagging predictors. Mach. Learn.*,* 24, 123–140.

[btv197-B4] Breiman L. (1996b) Heuristics of instability and stabilization in model selection. Ann. Stat.*,* 24, 2350–2383.

[btv197-B5] Breiman L. (2001) Random forests. Mach. Learn.*,* 45, 5–32.

[btv197-B6] Eckart C. Young G. (1936) The approximation of one matrix by another of lower rank. Psychometrika*,* 1, 211–218.

[btv197-B7] Fan J. Li R. (2001) Variable selection via nonconcave penalized likelihood and its oracle properties. J. Am. Stat. Assoc.*,* 96, 1348–1360.

[btv197-B8] Jolliffe I.T. (1982) A note on the use of principal components in regression. Appl. Stat.*,* 31, 300+.

[btv197-B9] Jolliffe I.T. (2003) A modified principal component technique based on the LASSO. J. Comput. Graph. Stat.*,* 12, 531–547.

[btv197-B10] Jung S. Marron J. (2009) PCA consistency in high dimension, low sample size context. Ann. Stat.*,* 37, 4104–4130.

[btv197-B11] Kanehisa M. Goto S. (2000) KEGG: Kyoto encyclopedia of genes and genomes. Nucleic Acids Res.*,* 28, 27–30. 10592173 10.1093/nar/28.1.27 PMC102409

[btv197-B12] Kauffmann A. (2009) arrayQualityMetrics: a bioconductor package for quality assessment of microarray data. Bioinformatics*,* 25, 415–416. 19106121 10.1093/bioinformatics/btn647 PMC2639074

[btv197-B13] Kim Y. (2012) Consistent model selection criteria on high dimensions. J. Mach. Learn. Res., 13, 1037–1057.

[btv197-B14] Lee M. (2010) Biclustering via sparse singular value decomposition. Biometrics*,* 66, 1087–1095. 20163403 10.1111/j.1541-0420.2010.01392.x

[btv197-B15] Ma S. Dai Y. (2011) Principal component analysis based methods in bioinformatics studies. Brief. Bioinf.*,* 12, 714–722. 10.1093/bib/bbq090 PMC3220871 21242203

[btv197-B16] Meinshausen N. Bühlmann P. (2006) High dimensional graphs and variable selection with the lasso. Ann. Stat.*,* 34, 1436–1462.

[btv197-B17] Meinshausen N. Bühlmann P. (2010) Stability selection. J. R. Stat. Soc. Ser. B*,* 72, 417–473.

[btv197-B18] Remke M. (2011) Fstl5 is a marker of poor prognosis in non-wnt/non-shh medulloblastoma. J. Clin. Oncol.*,* 29, 3852–3861. 21911727 10.1200/JCO.2011.36.2798

[btv197-B19] Shen D. (2013) Consistency of sparse pca in high dimension, low sample size contexts. J. Multivar. Anal.*,* 115, 317–333.

[btv197-B20] Shen H. Huang J. (2008) Sparse principal component analysis via regularized low rank matrix approximation. J. Multivar. Anal.*,* 99, 1015–1034.

[btv197-B21] Tibshirani R. (1996) Regression shrinkage and selection via the lasso. J. R. Stat. Soc. Ser. B*,* 58, 267–288.

[btv197-B22] Wang X. (2011) HTSanalyzeR: an R/Bioconductor package for integrated network analysis of high-throughput screens. Bioinformatics*,* 27, 879–880. 21258062 10.1093/bioinformatics/btr028 PMC3051329

[btv197-B23] Witten D.M. (2009) A penalized matrix decomposition, with applications to sparse principal components and canonical correlation analysis. Biostatistics*,* 10, 515–534. 19377034 10.1093/biostatistics/kxp008 PMC2697346

[btv197-B24] Yang D. (2014) A sparse singular value decomposition method for high-dimensional data. J. Comput. Graph. Stat.*,* 23, 923–942.

[btv197-B25] Zhao P. Yu B. (2006) On model selection consistency of lasso. J. Mach. Learn. Res.*,* 7, 2541–2563.

[btv197-B26] Zou H. (2006) The adaptive LASSO and its oracle properties. J. Am. Stat. Assoc.*,* 101, 1418–1429.

[btv197-B27] Zou H. (2004). Sparse principal component analysis. J. Comput. Graph. Stat.*,* 15, 1–30.

