# Supplementary material for: Applying stability selection to consistently estimate sparse principal components in high-dimensional molecular data
Source: Bioinformatics. 2015 Apr 10;31(16):2683–90. doi: 10.1093/bioinformatics/btv197 (PMC4528629; doi:10.1093/bioinformatics/btv197)
Supplement: Supplementary Data [file supp_31_16_2683__index.html]

Applying Stability Selection to Consistently Estimate Sparse Principal Components in High-Dimensional Molecular Data — Applying stability selection to consistently estimate sparse principal components in high-dimensional molecular data — Applying stability selection to consistently estimate sparse principal components in high-dimensional molecular data — Supplementary Data 

# Applying stability selection to consistently estimate sparse principal components in high-dimensional molecular data

## Supplementary Data

files

**Files in this Data Supplement:**

- Supplementary Data - pdf file
