# Supplementary material for: Applying stability selection to consistently estimate sparse principal components in high-dimensional molecular data
Source: Bioinformatics. 2015 Apr 10;31(16):2683–90. doi: 10.1093/bioinformatics/btv197 (PMC4528629; doi:10.1093/bioinformatics/btv197)
Supplement: Supplementary Data [file supp_btv197_supplementary.pdf]

Applying Stability Selection to Consistently Estimate Sparse Principal Components in High-Dimensional Molecular Data

Supplementary Material

1 Real Data Application

1.1 S4VDPCA

KEGG pathway	Gene.Set.Size	Expected.Hits	Observed.Hits	Pvalue	Adjusted.Pvalue
PC1					
Ribosome	47.00	6.08	38.00	0.00	0.00
Basal cell carcinoma	39.00	5.05	16.00	0.00	0.00
Neuroactive ligand-receptor interaction	121.00	15.66	30.00	0.00	0.01
Dilated cardiomyopathy	66.00	8.54	19.00	0.00	0.02
Hypertrophic cardiomyopathy (HCM)	61.00	7.89	17.00	0.00	0.04
Hedgehog signaling pathway	36.00	4.66	12.00	0.00	0.04
Arrhythmogenic right ventricular cardiomyopathy (ARVC)	52.00	6.73	15.00	0.00	0.04
Pathways in cancer	259.00	33.52	50.00	0.00	0.04
Wnt signaling pathway	119.00	15.40	27.00	0.00	0.04
Calcium signaling pathway	123.00	15.92	27.00	0.00	0.06
PC2					
Basal cell carcinoma	39.00	3.86	16.00	0.00	0.00
Wnt signaling pathway	119.00	11.78	29.00	0.00	0.00
Hedgehog signaling pathway	36.00	3.56	12.00	0.00	0.01
Melanogenesis	78.00	7.72	19.00	0.00	0.01
ECM-receptor interaction	57.00	5.64	15.00	0.00	0.01
Axon guidance	107.00	10.59	22.00	0.00	0.02
Pathways in cancer	259.00	25.64	41.00	0.00	0.04
Calcium signaling pathway	123.00	12.18	23.00	0.00	0.04
Neuroactive ligand-receptor interaction	121.00	11.98	22.00	0.00	0.06
Cytokine-cytokine receptor interaction	113.00	11.19	20.00	0.01	0.11

Table 1: First ten KEGG pathways most significantly overrepresented by genes selected for the first and the second sparse PC estimated by S4VDPCA. Gene sets have been tested by hypergeometric tests as implemented in the Bioconductor-package *HTSanalyzeR*. P-values have been adjusted for multiple testing by applying the Benjamini-Hochberg procedure.

	angle
angle(pc1,pc2)	89.98
angle(pc1,pc3)	89.97
angle(pc2,pc3)	89.57

Table 2: Pairwise angles between the first three sparse PCs estimated by S4VPCA.

	emp_spike	emp_sparsity
pc1	0.73	0.78
pc2	0.69	0.75

Table 3: Empirical spike index ($\hat{\alpha}$) and empirical sparsity index ($\hat{\beta}$) for the first two sparse PCs estimated by S4VDPCA.

1.2 RSPCA lasso

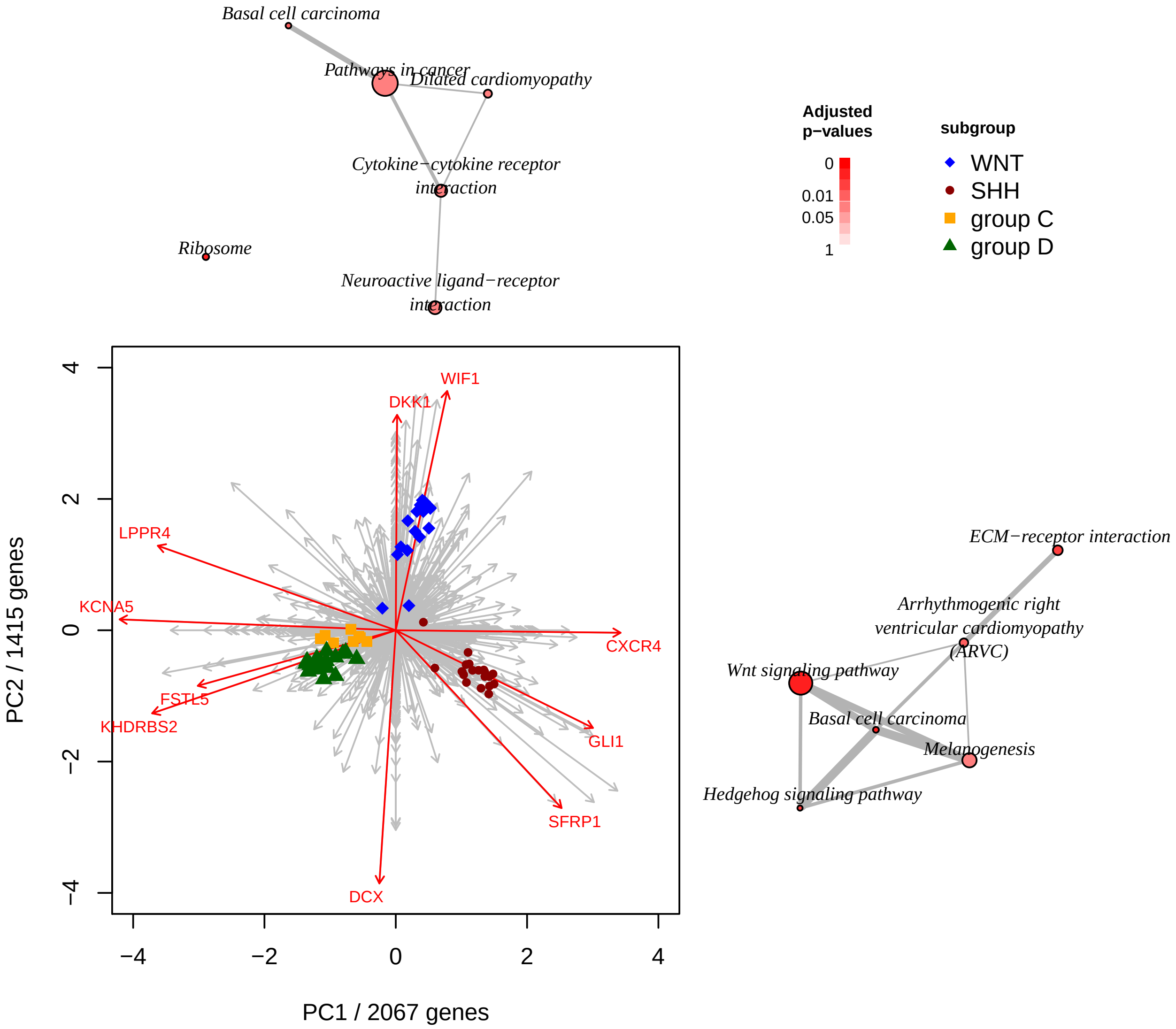

Figure 1: Biplot representation of the first two sparse PCs estimated by RSPCA lasso. The biplot displays the projection of the samples into the two dimensional space spanned by the first two sparse PCs. The arrows show the contribution of the selected genes to the two sparse PCs, i.e. the covariance structure of the selected genes. Each arrow represents a gene and the length of the arrow reflects the size of the corresponding coefficient in the two loadings vectors. Relevant oncogenes are highlighted in red. The nodes of the two graphs above and on the right side of the biplot represent pathways significantly overrepresented by the genes selected in the first and second PC, respectively.

KEGG pathway	Gene.Set.Size	Expected.Hits	Observed.Hits	Pvalue	Adjusted.Pvalue
PC1					
Ribosome	47.00	6.11	39.00	0.00	0.00
Basal cell carcinoma	39.00	5.07	15.00	0.00	0.00
Neuroactive ligand-receptor interaction	121.00	15.73	30.00	0.00	0.02
Dilated cardiomyopathy	66.00	8.58	19.00	0.00	0.02
Pathways in cancer	259.00	33.68	51.00	0.00	0.03
Cytokine-cytokine receptor interaction	113.00	14.69	27.00	0.00	0.03
Hypertrophic cardiomyopathy (HCM)	61.00	7.93	17.00	0.00	0.03
Amyotrophic lateral sclerosis (ALS)	45.00	5.85	14.00	0.00	0.03
Calcium signaling pathway	123.00	15.99	27.00	0.00	0.07
Hedgehog signaling pathway	36.00	4.68	11.00	0.00	0.08
PC2					
Basal cell carcinoma	39.00	3.54	16.00	0.00	0.00
Wnt signaling pathway	119.00	10.79	27.00	0.00	0.00
ECM-receptor interaction	57.00	5.17	16.00	0.00	0.00
Hedgehog signaling pathway	36.00	3.27	12.00	0.00	0.00
Arrhythmogenic right ventricular cardiomyopathy (ARVC)	52.00	4.72	14.00	0.00	0.01
Melanogenesis	78.00	7.07	17.00	0.00	0.01
Pathways in cancer	259.00	23.49	39.00	0.00	0.03
Axon guidance	107.00	9.71	20.00	0.00	0.03
Amoebiasis	70.00	6.35	14.00	0.00	0.07
Dilated cardiomyopathy	66.00	5.99	13.00	0.01	0.08

Table 4: First ten KEGG pathways most significantly overrepresented by genes selected for the first and the second sparse PC estimated by RSPCA lasso. Gene sets have been tested by hypergeometric tests as implemented in the Bioconductor-package *HTSanalyzeR*. P-values have been adjusted for multiple testing by applying the Benjamini-Hochberg procedure.

	angle
angle(pc1,pc2)	88.97
angle(pc1,pc3)	88.58
angle(pc2,pc3)	89.75

Table 5: Pairwise angles between the first three sparse PCs estimated by RSPCA lasso.

	emp_spike	emp_sparsity
pc1	0.71	0.78
pc2	0.67	0.74

Table 6: Empirical spike index ($\hat{\alpha}$) and empirical sparsity index ($\hat{\beta}$) for the first two sparse PCs estimated by RSPCA lasso.

1.3 RSPCA adaptive lasso

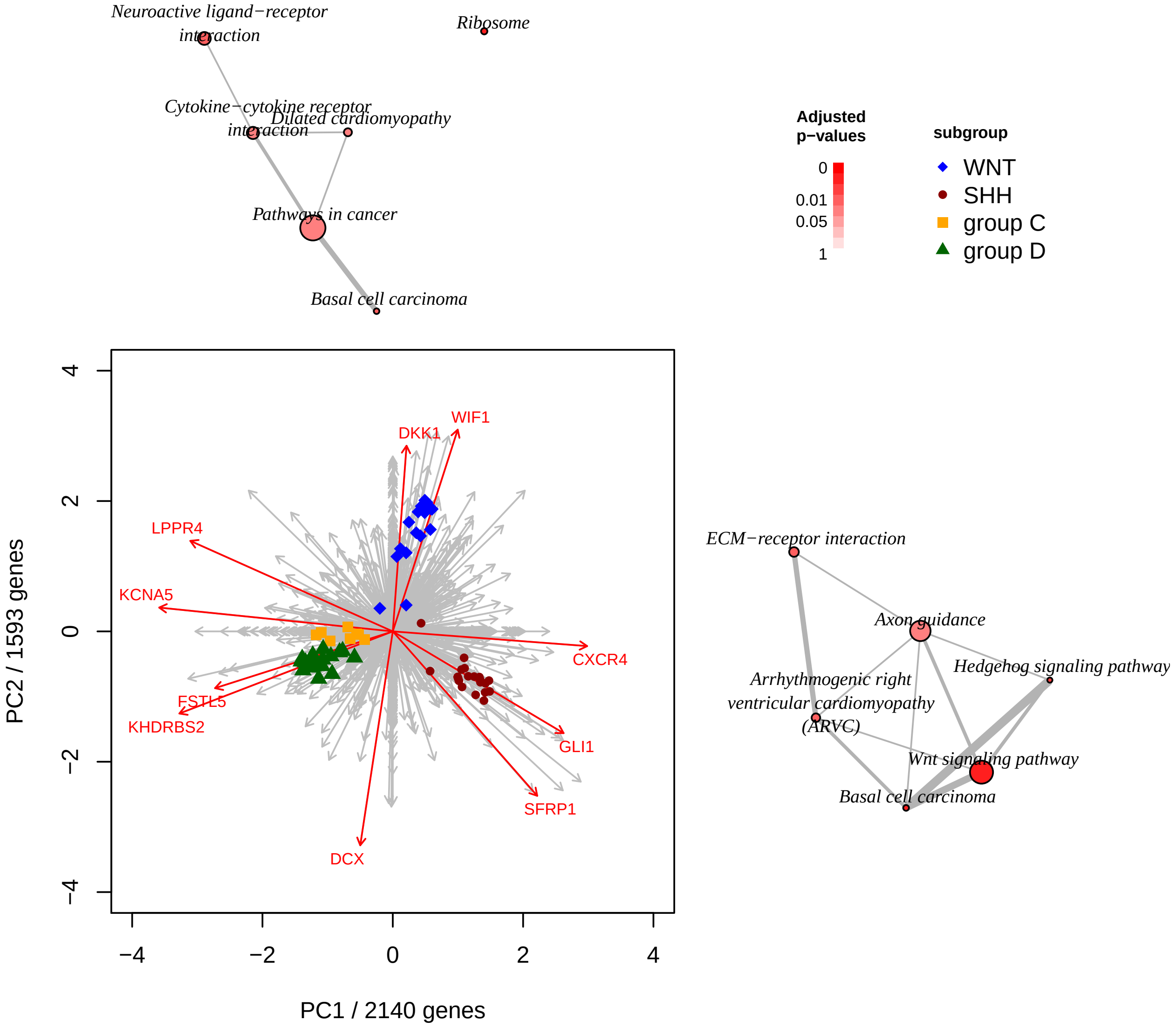

Figure 2: Biplot representation of the first two sparse PCs estimated by RSPCA adaptive lasso. The biplot displays the projection of the samples into the two dimensional space spanned by the first two sparse PCs. The arrows show the contribution of the selected genes to the two sparse PCs, i.e. the covariance structure of the selected genes. Each arrow represents a gene and the length of the arrow reflects the size of the corresponding coefficient in the two loadings vectors. Relevant oncogenes are highlighted in red. The nodes of the two graphs above and on the right side of the biplot represent pathways significantly overrepresented by the genes selected in the first and second PC, respectively.

KEGG pathway	Gene.Set.Size	Expected.Hits	Observed.Hits	Pvalue	Adjusted.Pvalue
PC1					
Ribosome	47.00	6.33	39.00	0.00	0.00
Neuroactive ligand-receptor interaction	121.00	16.29	32.00	0.00	0.01
Basal cell carcinoma	39.00	5.25	15.00	0.00	0.01
Cytokine-cytokine receptor interaction	113.00	15.21	29.00	0.00	0.01
Dilated cardiomyopathy	66.00	8.88	19.00	0.00	0.03
Pathways in cancer	259.00	34.86	53.00	0.00	0.03
Hypertrophic cardiomyopathy (HCM)	61.00	8.21	17.00	0.00	0.05
Amyotrophic lateral sclerosis (ALS)	45.00	6.06	13.00	0.01	0.10
Calcium signaling pathway	123.00	16.56	27.00	0.01	0.10
Hedgehog signaling pathway	36.00	4.85	11.00	0.01	0.10
PC2					
Basal cell carcinoma	39.00	3.98	16.00	0.00	0.00
Wnt signaling pathway	119.00	12.15	29.00	0.00	0.00
Hedgehog signaling pathway	36.00	3.68	13.00	0.00	0.00
Arrhythmogenic right ventricular cardiomyopathy (ARVC)	52.00	5.31	15.00	0.00	0.00
ECM-receptor interaction	57.00	5.82	16.00	0.00	0.00
Axon guidance	107.00	10.93	22.00	0.00	0.03
Pathways in cancer	259.00	26.45	42.00	0.00	0.04
Melanogenesis	78.00	7.97	17.00	0.00	0.04
Calcium signaling pathway	123.00	12.56	23.00	0.00	0.05
Dilated cardiomyopathy	66.00	6.74	14.00	0.01	0.10

Table 7: First ten KEGG pathways most significantly overrepresented by genes selected for the first and the second sparse PC estimated by RSPCA adaptive lasso. Gene sets have been tested by hypergeometric tests as implemented in the Bioconductor-package *HTSanalyzeR*. P-values have been adjusted for multiple testing by applying the Benjamini-Hochberg procedure.

	angle
angle(pc1,pc2)	89.31
angle(pc1,pc3)	89.24
angle(pc2,pc3)	90.00

Table 8: Pairwise angles between the first three sparse PCs estimated by RSPCA adaptive lasso.

	emp_spike	emp_sparsity
pc1	0.72	0.78
pc2	0.68	0.75

Table 9: Empirical spike index ($\hat{\alpha}$) and empirical sparsity index ($\hat{\beta}$) for the first two sparse PCs estimated by RSPCA adaptive lasso.

1.4 RSPCA SCAD

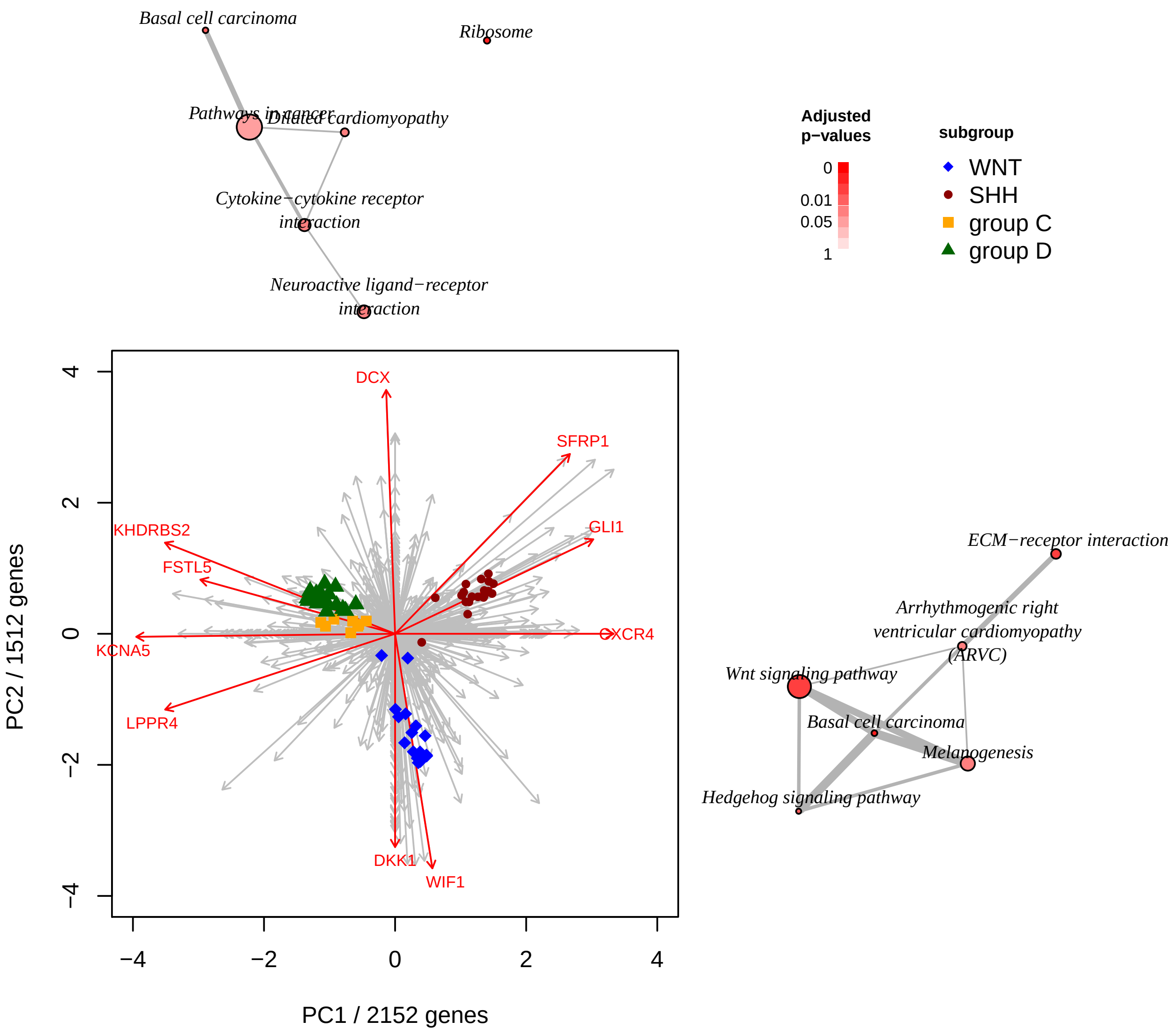

Figure 3: Biplot representation of the first two sparse PCs estimated by RSPCA SCAD. The biplot displays the projection of the samples into the two dimensional space spanned by the first two sparse PCs. The arrows show the contribution of the selected genes to the two sparse PCs, i.e. the covariance structure of the selected genes. Each arrow represents a gene and the length of the arrow reflects the size of the corresponding coefficient in the two loadings vectors. Relevant oncogenes are highlighted in red. The nodes of the two graphs above and on the right side of the biplot represent pathways significantly overrepresented by the genes selected in the first and second PC, respectively.

KEGG pathway	Gene.Set.Size	Expected.Hits	Observed.Hits	Pvalue	Adjusted.Pvalue
PC1					
Ribosome	47.00	6.33	39.00	0.00	0.00
Neuroactive ligand-receptor interaction	121.00	16.29	32.00	0.00	0.01
Basal cell carcinoma	39.00	5.25	15.00	0.00	0.01
Cytokine-cytokine receptor interaction	113.00	15.21	29.00	0.00	0.01
Dilated cardiomyopathy	66.00	8.88	19.00	0.00	0.03
Pathways in cancer	259.00	34.86	53.00	0.00	0.03
Hypertrophic cardiomyopathy (HCM)	61.00	8.21	17.00	0.00	0.05
Amyotrophic lateral sclerosis (ALS)	45.00	6.06	13.00	0.01	0.10
Calcium signaling pathway	123.00	16.56	27.00	0.01	0.10
Hedgehog signaling pathway	36.00	4.85	11.00	0.01	0.10
PC2					
Basal cell carcinoma	39.00	3.98	16.00	0.00	0.00
Wnt signaling pathway	119.00	12.15	29.00	0.00	0.00
Hedgehog signaling pathway	36.00	3.68	13.00	0.00	0.00
Arrhythmogenic right ventricular cardiomyopathy (ARVC)	52.00	5.31	15.00	0.00	0.00
ECM-receptor interaction	57.00	5.82	16.00	0.00	0.00
Axon guidance	107.00	10.93	22.00	0.00	0.03
Pathways in cancer	259.00	26.45	42.00	0.00	0.04
Melanogenesis	78.00	7.97	17.00	0.00	0.04
Calcium signaling pathway	123.00	12.56	23.00	0.00	0.05
Dilated cardiomyopathy	66.00	6.74	14.00	0.01	0.10

Table 10: First ten KEGG pathways most significantly overrepresented by genes selected for the first and the second sparse PC estimated by RSPCA SCAD. Gene sets have been tested by hypergeometric tests as implemented in the Bioconductor-package *HTSanalyzeR*. P-values have been adjusted for multiple testing by applying the Benjamini-Hochberg procedure.

	angle
angle(pc1,pc2)	88.65
angle(pc1,pc3)	88.78
angle(pc2,pc3)	89.84

Table 11: Pairwise angles between the first three sparse PCs estimated by RSPCA SCAD.

	emp_spike	emp_sparsity
pc1	0.70	0.78
pc2	0.67	0.75

Table 12: Empirical spike index ($\hat{\alpha}$) and empirical sparsity index ($\hat{\beta}$) for the first two sparse PCs estimated by RSPCA SCAD.

1.5 Comparison

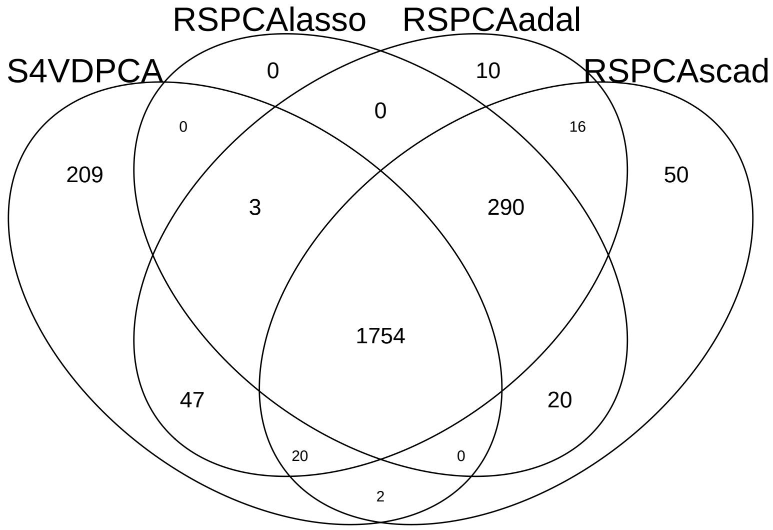

Figure 4: Venn diagram that displays the agreement between the first sparse PCs estimated by the different sparse PCA methods.

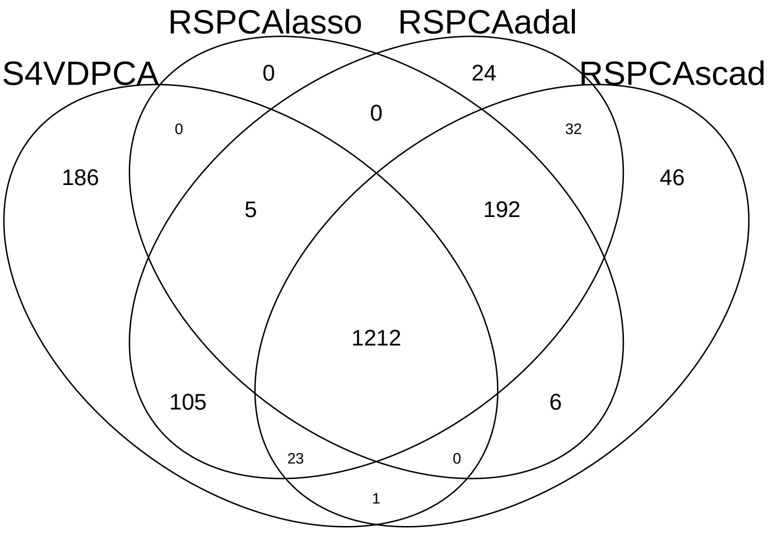

Figure 5: Venn diagram that displays the agreement between the second sparse PCs estimated by the different sparse PCA methods.

1.6 PCA

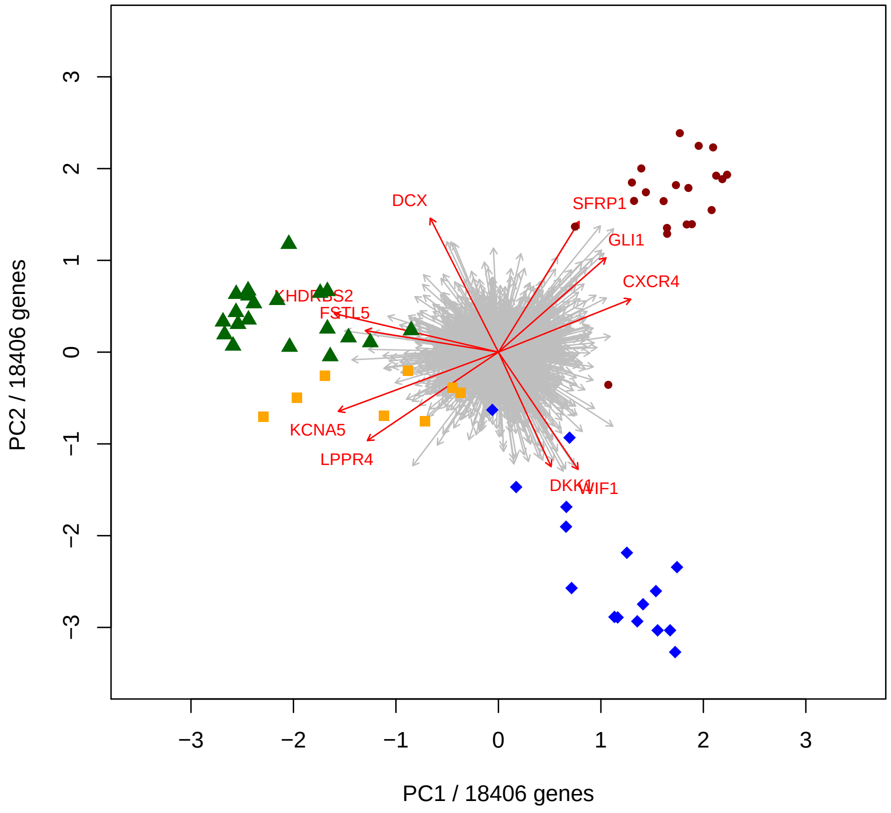

Figure 6: Biplot representation of the first two PCs estimated by conventional PCA. The biplot displays the projection of the samples into the two dimensional space spanned by the first two PCs. The arrows show the contribution of the selected genes to the two PCs, i.e. the covariance structure of the selected genes. Each arrow represents a gene and the length of the arrow reflects the size of the corresponding coefficient in the two loadings vectors. Relevant oncogenes are highlighted in red.

1.7 Screeplot

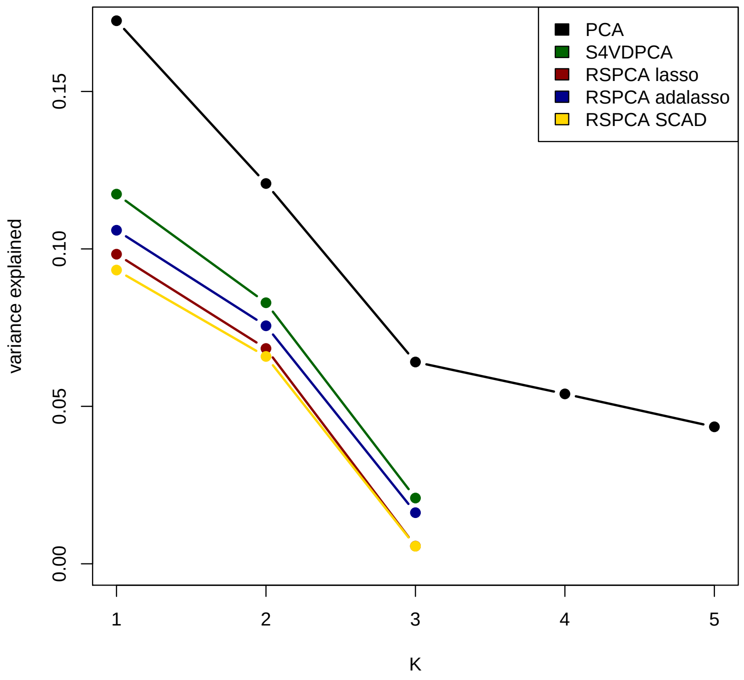

Figure 7: The screeplot shows the amount of explained variation of the first few PCs estimated by conventional PCA and different sparse PCA methods.

2 Additional Simulations

2.1 Angle for additional parameter settings

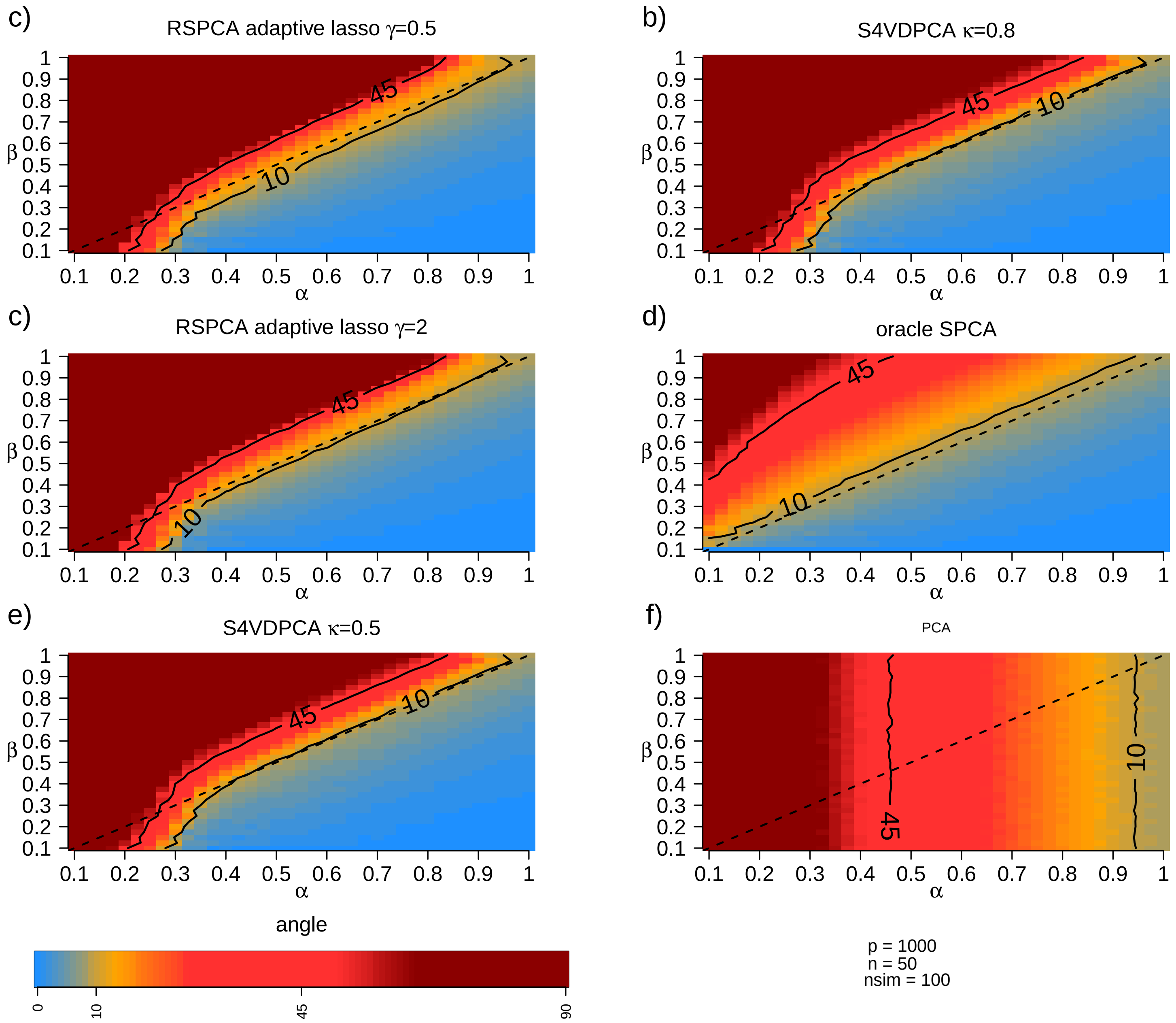

Figure 8: Angles between estimated and true leading eigenvector for a) RSPCA with adaptive *lasso* penalty ($\gamma = 0.5$), b) S4VDPCA with $\kappa = 0.8$, c) RSPCA with adaptive *lasso* penalty ($\gamma = 2$), d) *oracle* SPCA, e) S4VDPCA with $\kappa = 0.5$ and f) conventional PCA. The colors correspond to the median angle calculated over 100 simulation runs. Angles with 10 and 45 degrees of deviation are indicated by contour lines. The sparsity index β and the spike index α define the sparsity, e.g. the number of truly non-zero coefficients, and the dominance of the signal, e.g. the eigenvalue, of the simulated first PC. Further, p and n denote the number of features and samples of the simulated data sets and *nsim* denotes the number of simulated data sets.

2.2 FDR for additional parameter settings

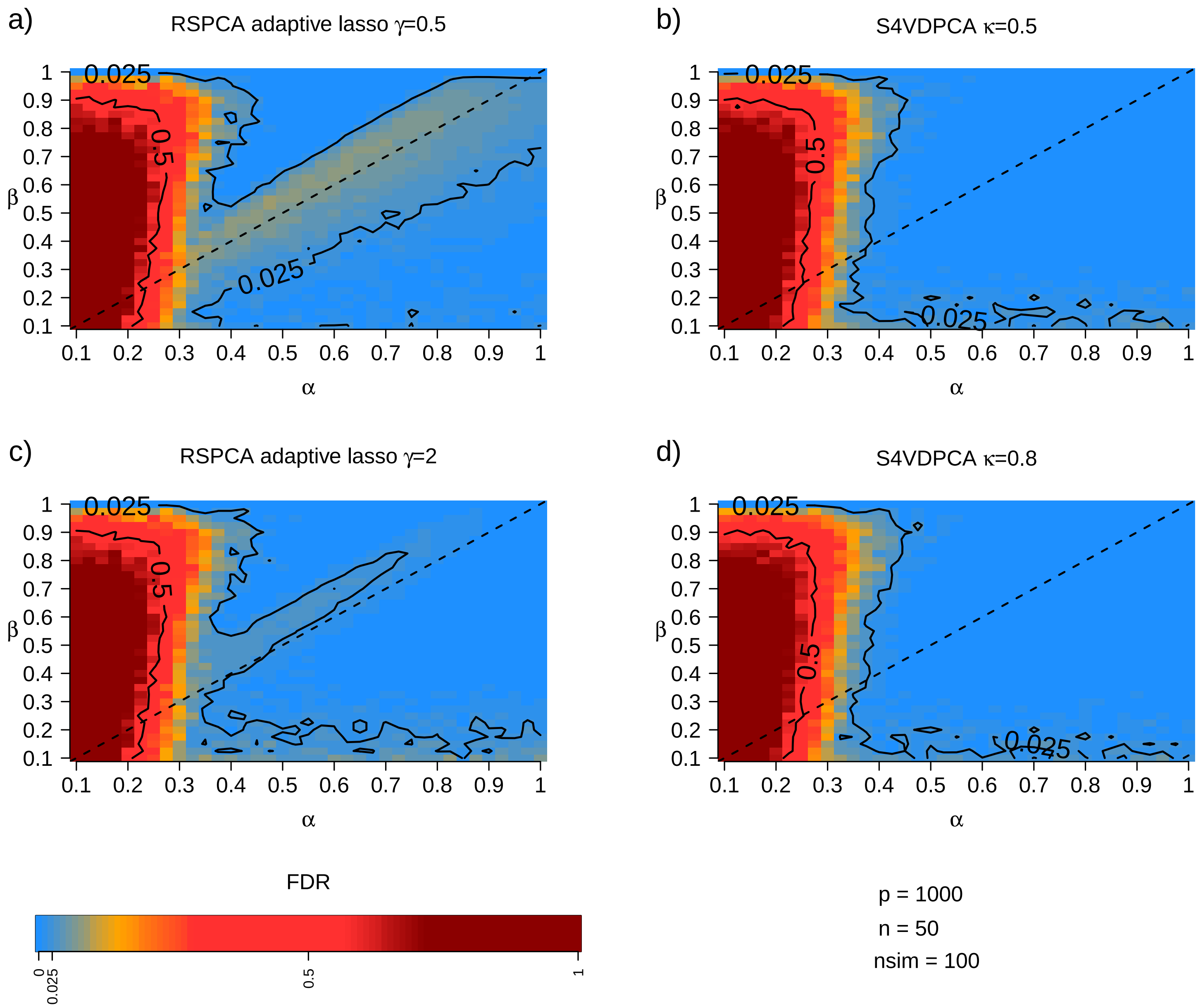

Figure 9: Median FDR for a) RSPCA with adaptive *lasso* penalty ($\gamma = 0.5$),b) S4VDPCA with $\kappa = 0.8$, c) RSPCA with adaptive *lasso* penalty ($\gamma = 2$) and d) S4VDPCA with $\kappa = 0.8$. FDRs of 0.05 and 0.5 are indicated by contour lines. The sparsity index β and the spike index α define the sparsity, e.g. the number of truely non-zero coefficients, and the dominance of the signal, e.g. the eigenvalue, of the simulated first PC. Further, p and n denote the number of features and samples of the simulated data sets and $nsim$ denotes the number of simulated data sets.

2.3 True positive rate (TPR/Recall)

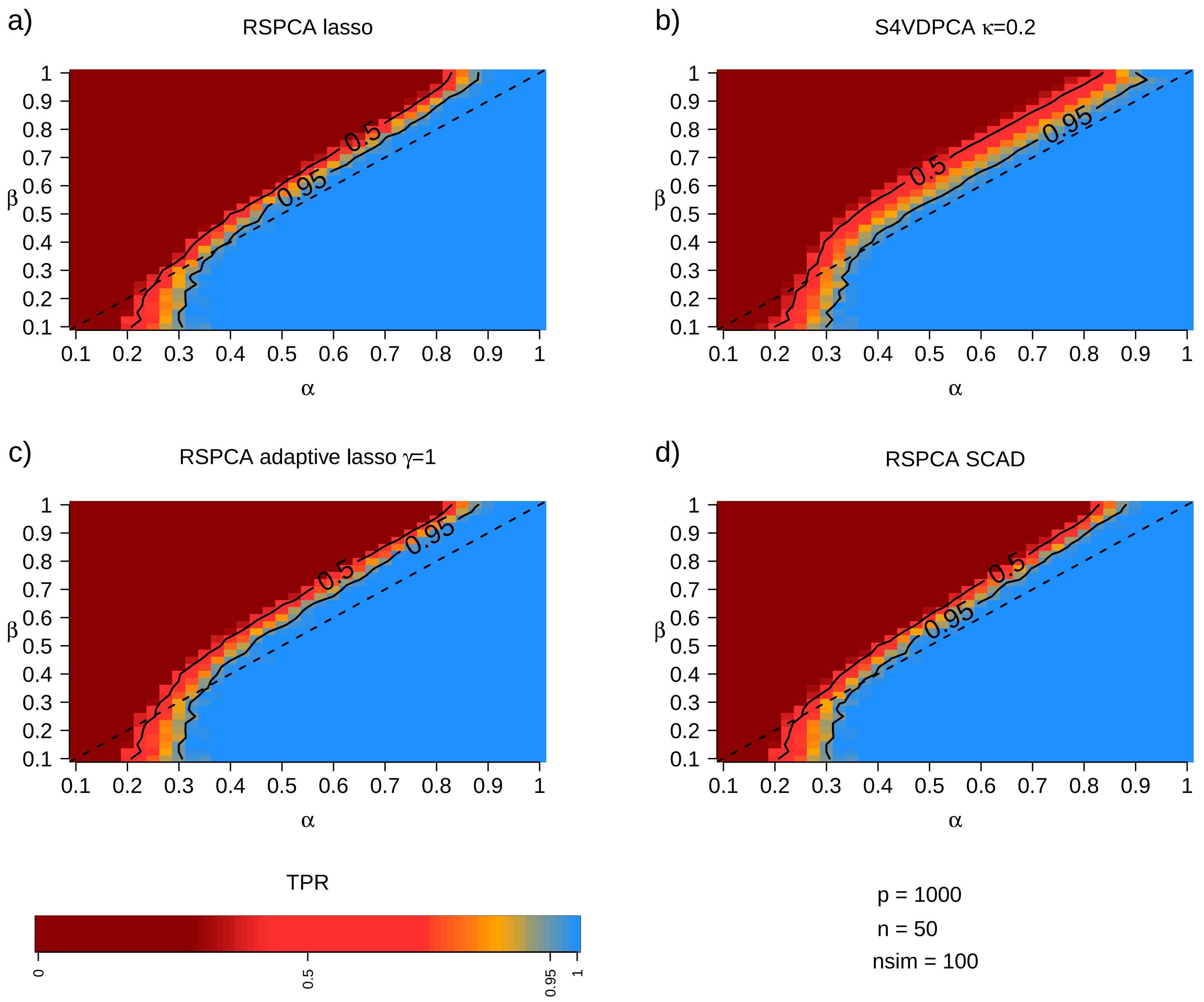

Figure 10: Median TPR for a) RSPCA with *lasso* penalty,b) S4VDPCA, c) RSPCA with adaptive *lasso* penalty and d) RSPCA with SCAD penalty. TPRs of 0.95 and 0.5 are indicated by contour lines. The sparsity index β and the spike index α define the sparsity, e.g. the number of truely non-zero coefficients, and the dominance of the signal, e.g. the eigenvalue, of the simulated first PC. Further, p and n denote the number of features and samples of the simulated data sets and $nsim$ denotes the number of simulated data sets.

2.4 TPR for additional parameter settings

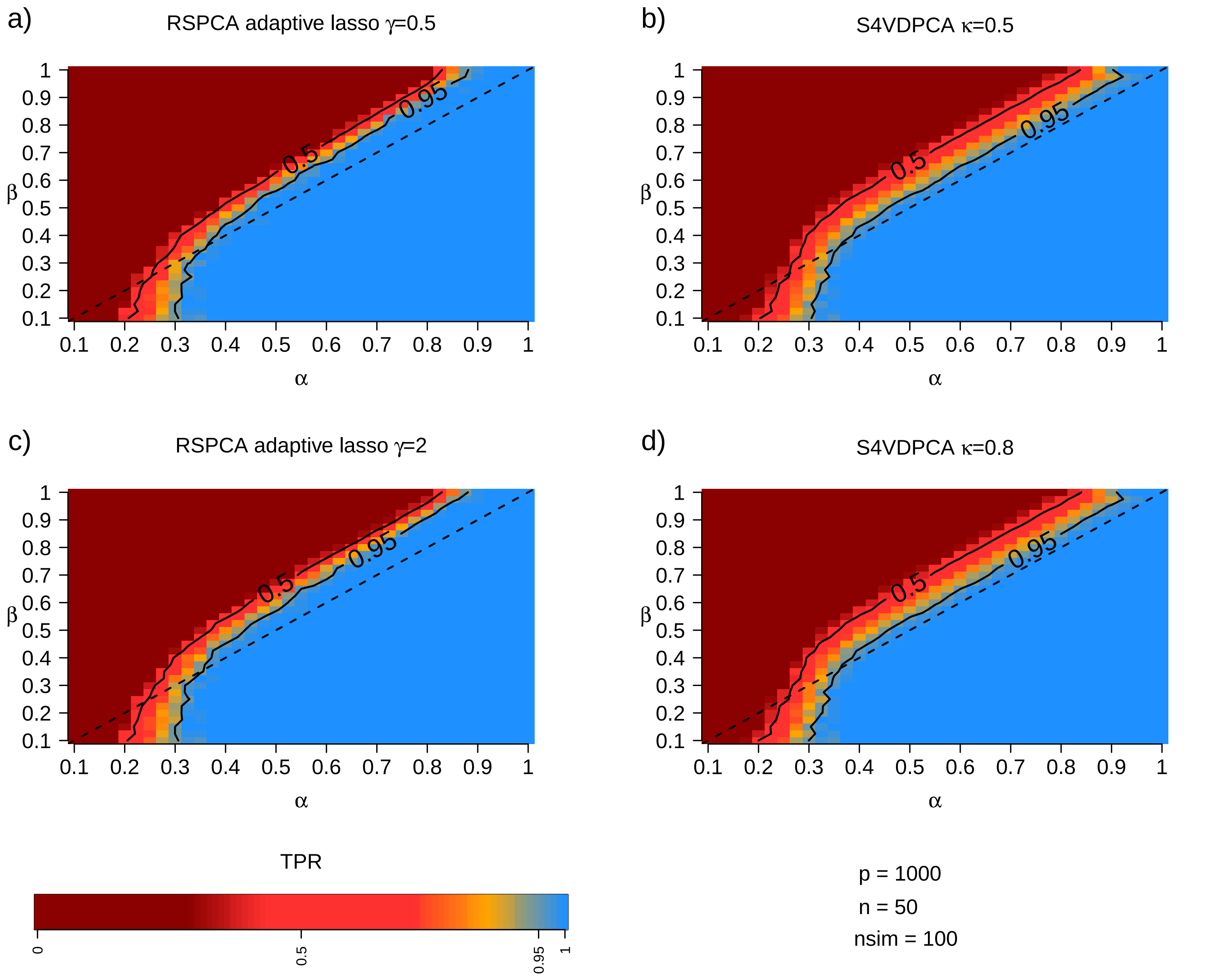

Figure 11: Median TPR for a) RSPCA with adaptive *lasso* penalty ($\gamma = 0.5$),b) S4VDPCA with $\kappa = 0.8$, c) RSPCA with adaptive *lasso* penalty ($\gamma = 2$) and d) S4VDPCA with $\kappa = 0.8$. TPRs of 0.95 and 0.5 are indicated by contour lines. The sparsity index β and the spike index α define the sparsity, e.g. the number of truely non-zero coefficients, and the dominance of the signal, e.g. the eigenvalue, of the simulated first PC. Further, p and n denote the number of features and samples of the simulated data sets and $nsim$ denotes the number of simulated data sets.

2.5 Empirical spike index

Given an estimated sparse PC the empirical spike index $\hat{\alpha}$ can be calculated by:

$$\hat{\alpha} = \frac{log(\frac{\hat{d}}{\sqrt{1-n}})^2}{log(p)}, \tag{1}$$

where $\hat{d}$ is the estimated singular value of the sparse PC.

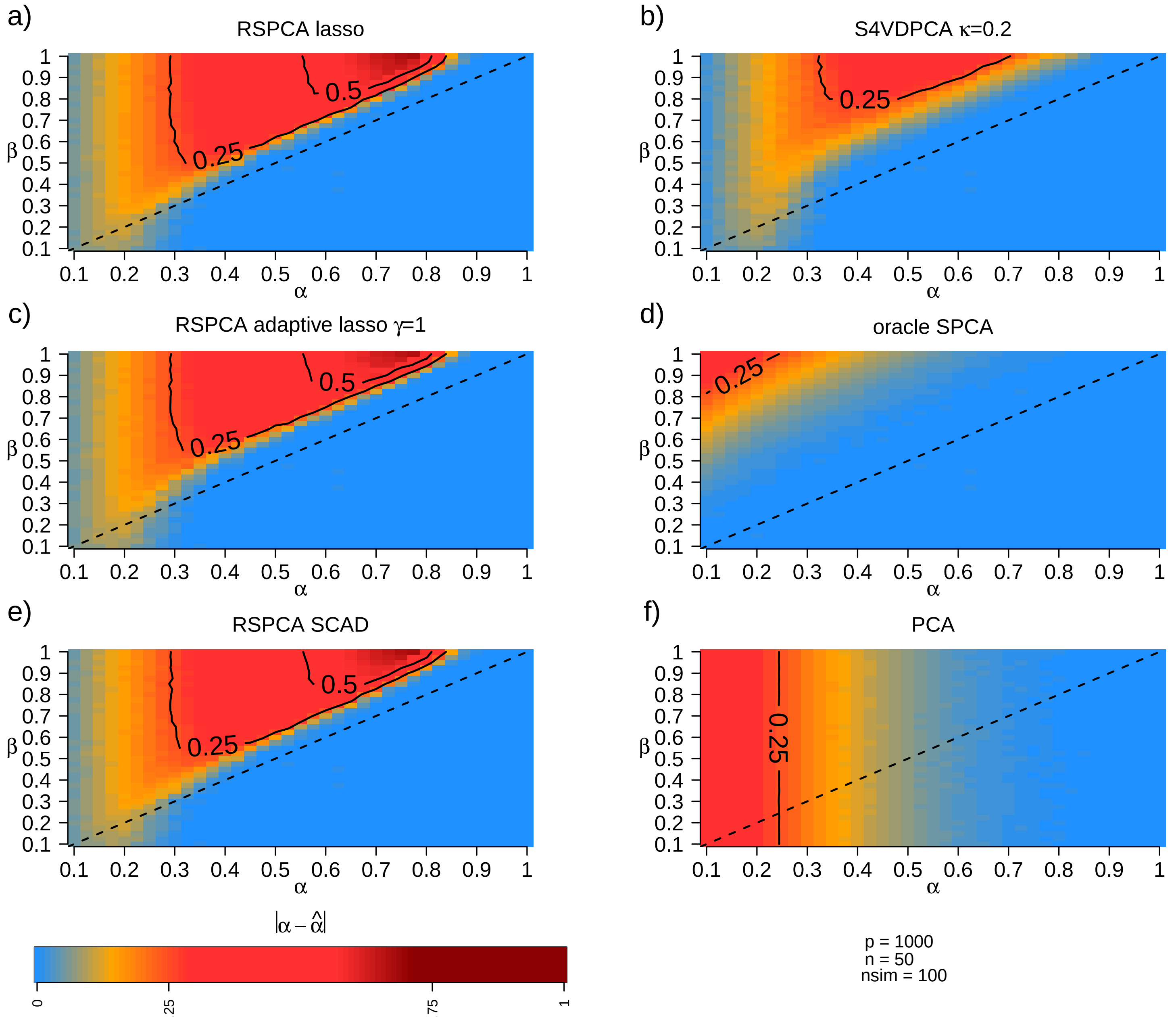

Figure 12: Absolute differences between empirical spike index and the simulated spike index for a) RSPCA with *lasso* penalty, b) S4VDPCA, c) RSPCA with adaptive *lasso* penalty, d) *oracle* SPCA, e) RSPCA with SCAD penalty and f) conventional PCA. The colors correspond to the median absolute differences calculated over 100 simulation runs. Absolute differences of 0.5 and 0.25 are indicated by contour lines. The sparsity index β and the spike index α define the sparsity, e.g. the number of truly non-zero coefficients, and the dominance of the signal, e.g. the eigenvalue, of the simulated first PC. Further, p and n denote the number of features and samples of the simulated data sets and $nsim$ denotes the number of simulated data sets.

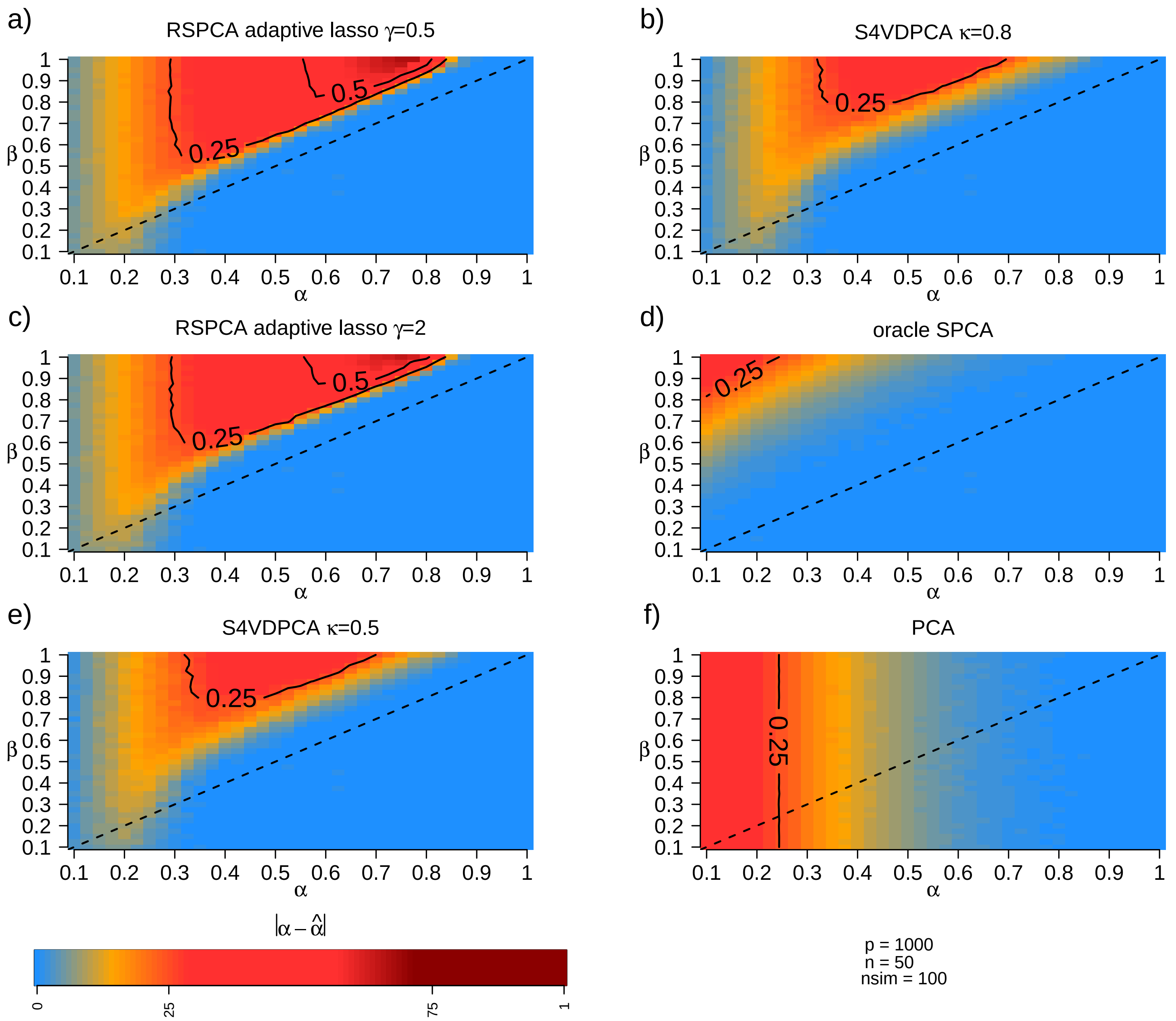

Figure 13: Absolute differences between empirical spike index and the simulated spike index for a) RSPCA with adaptive *lasso* penalty ($\gamma = 0.5$), b) S4VDPCA with $\kappa = 0.8$, c) RSPCA with adaptive *lasso* penalty ($\gamma = 2$), d) *oracle* SPCA, e) S4VDPCA with $\kappa = 0.5$ and f) conventional PCA. The colors correspond to the median absolute differences calculated over 100 simulation runs. Absolute differences of 0.5 and 0.25 are indicated by contour lines. The sparsity index β and the spike index α define the sparsity, e.g. the number of truly non-zero coefficients, and the dominance of the signal, e.g. the eigenvalue, of the simulated first PC. Further, p and n denote the number of features and samples of the simulated data sets and *nsim* denotes the number of simulated data sets.

2.6 Empirical sparsity index

Given an estimated sparse PC the empirical spike index $\hat{\beta}$ can be calculated by:

$$\hat{\beta} = \frac{log(|\hat{\mathbf{v}} \neq 0|)}{log(p)} \quad (2)$$

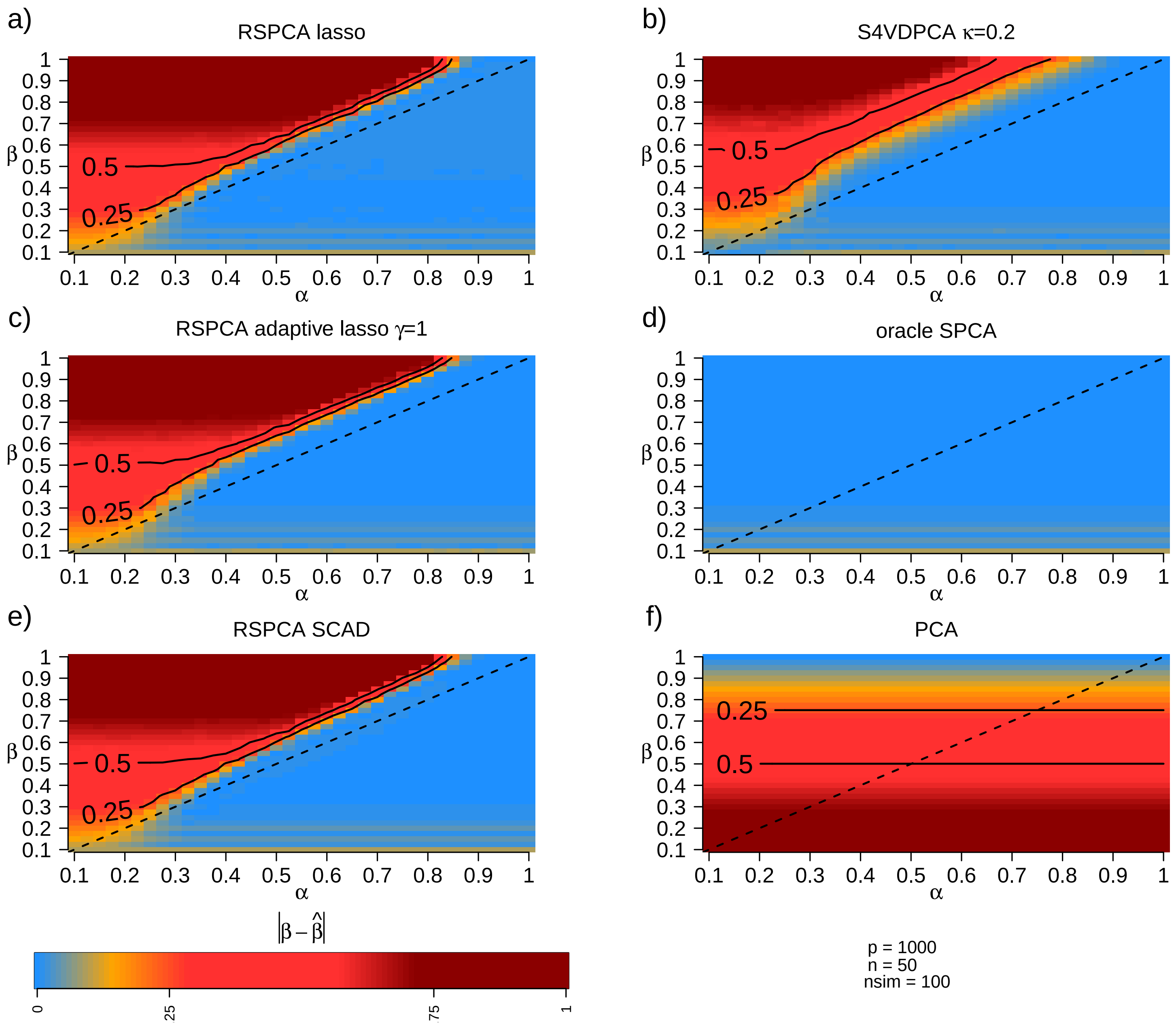

Figure 14: Absolute differences between empirical sparsity index and the simulated sparsity index for a) RSPCA with *lasso* penalty, b) S4VDPCA, c) RSPCA with adaptive *lasso* penalty, d) *oracle* SPCA, e) RSPCA with SCAD penalty and f) conventional PCA. The colors correspond to the median absolute differences calculated over 100 simulation runs. Absolute differences of 0.5 and 0.25 are indicated by contour lines. The sparsity index β and the spike index α define the sparsity, e.g. the number of truly non-zero coefficients, and the dominance of the signal, e.g. the eigenvalue, of the simulated first PC. Further, p and n denote the number of features and samples of the simulated data sets and $nsim$ denotes the number of simulated data sets.

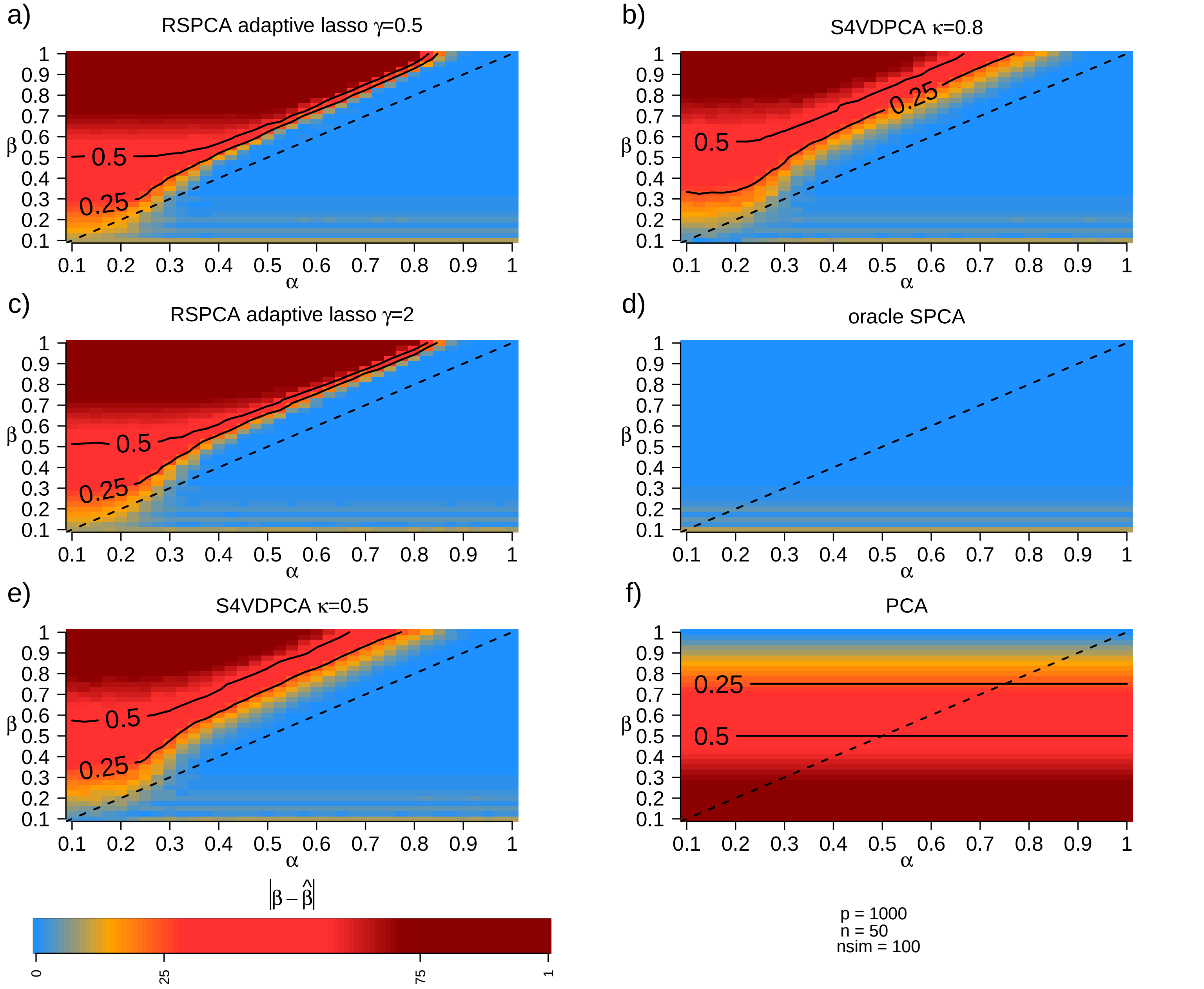

Figure 15: Absolute differences between empirical sparsity index and the simulated sparsity index for a) RSPCA with adaptive *lasso* penalty ($\gamma = 0.5$), b) S4VDPCA with $\kappa = 0.8$, c) RSPCA with adaptive *lasso* penalty ($\gamma = 2$), d) *oracle* SPCA, e) S4VDPCA with $\kappa = 0.5$ and f) conventional PCA. The colors correspond to the median absolute differences calculated over 100 simulation runs. Absolute differences of 0.5 and 0.25 are indicated by contour lines. The sparsity index β and the spike index α define the sparsity, e.g. the number of truly non-zero coefficients, and the dominance of the signal, e.g. the eigenvalue, of the simulated first PC. Further, p and n denote the number of features and samples of the simulated data sets and *nsim* denotes the number of simulated data sets.

3 Detailed simulation results for different combinations of simulated spike indices α and sparsity indices β

3.1 $\alpha = 0.8$ $\beta = 0.3$

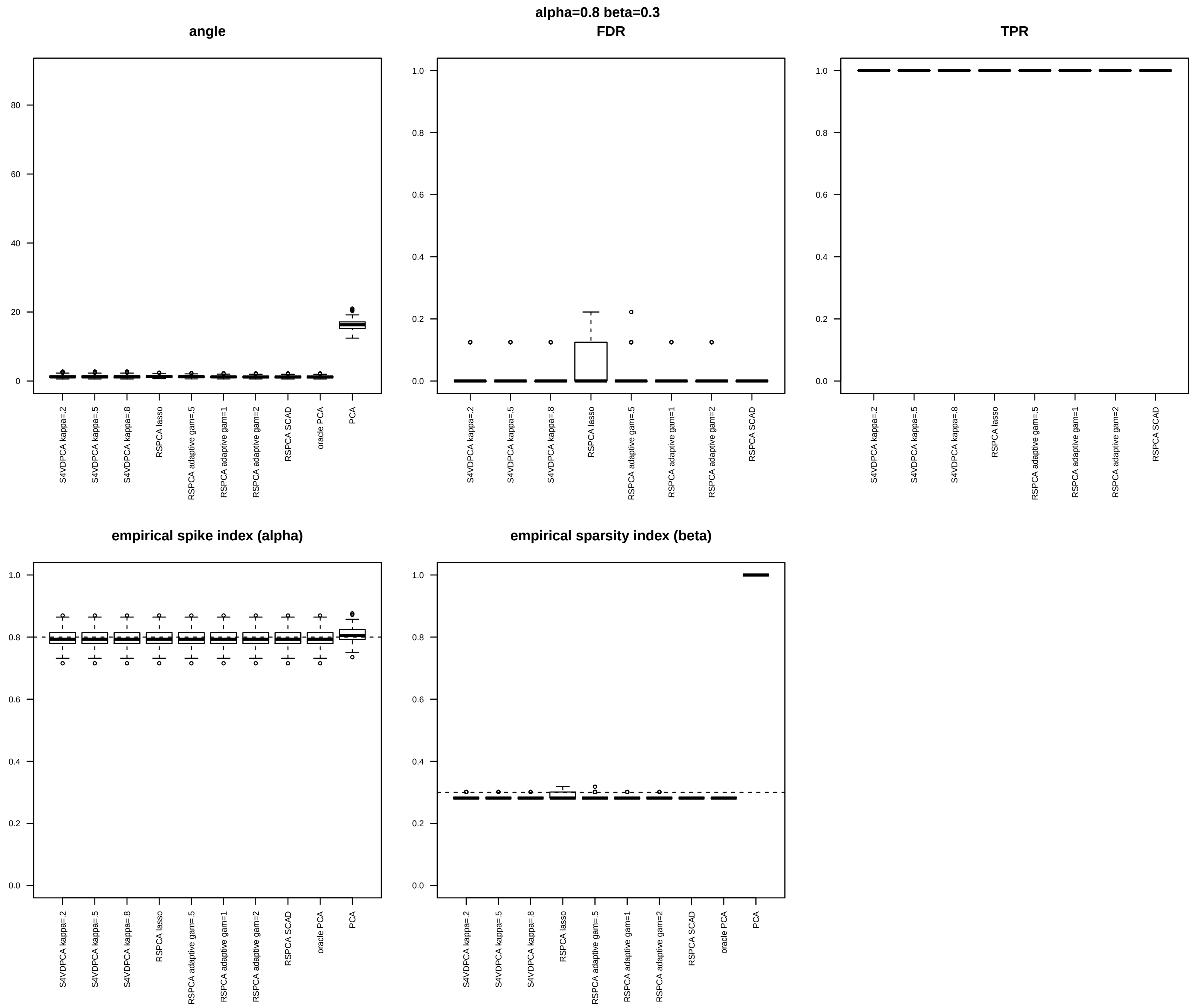

Figure 16: The boxplots display the angle between estimated and true leading eigenvector, FDR, TPR, empirical spike index and empirical sparsity index for 100 simulation runs.

3.2 $\alpha = 0.8$ $\beta = 0.8$

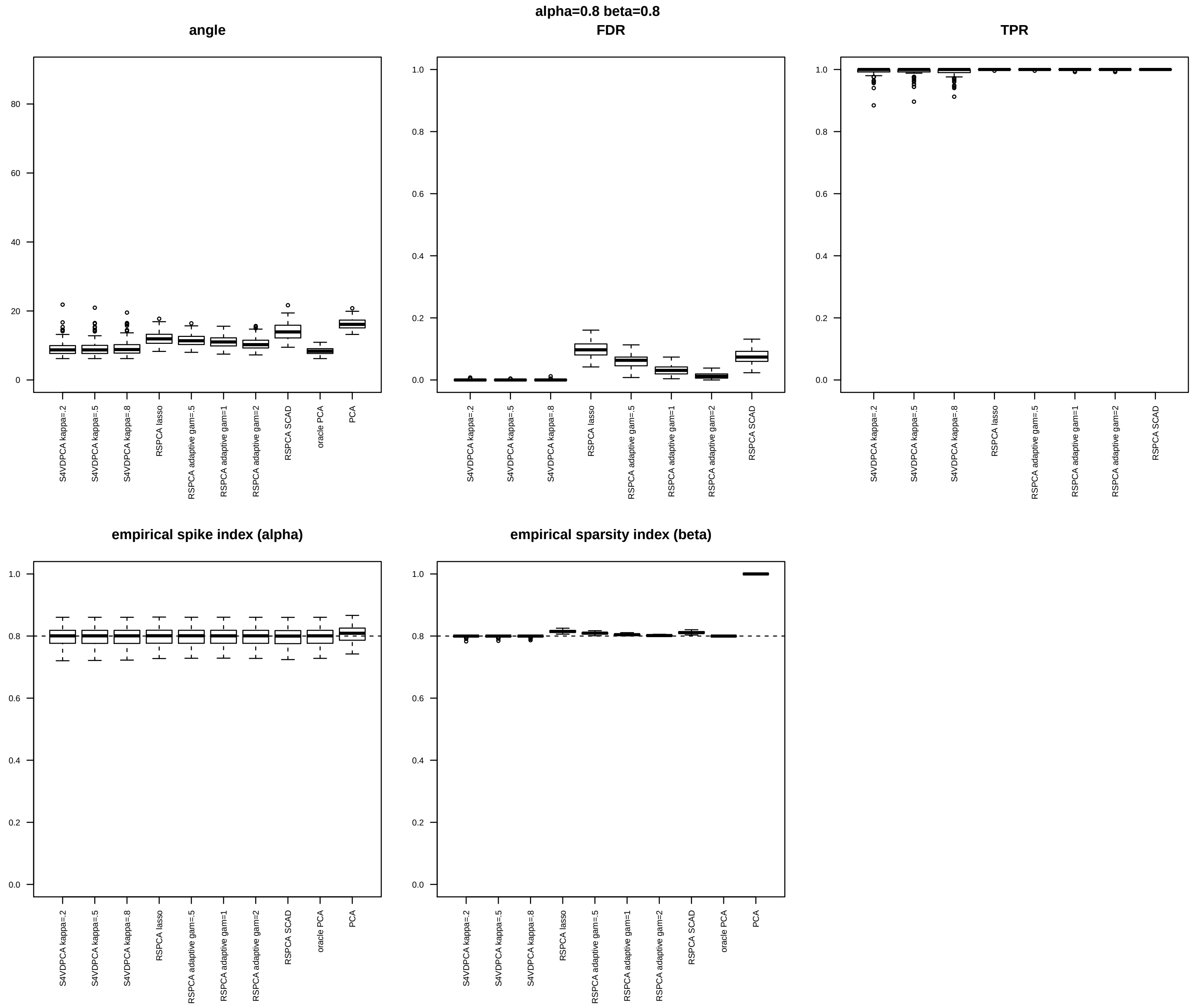

Figure 17: The boxplots display the angle between estimated and true leading eigenvector, FDR, TPR, empirical spike index and empirical sparsity index for 100 simulation runs.

3.3 $\alpha = 0.4$ $\beta = 0.4$

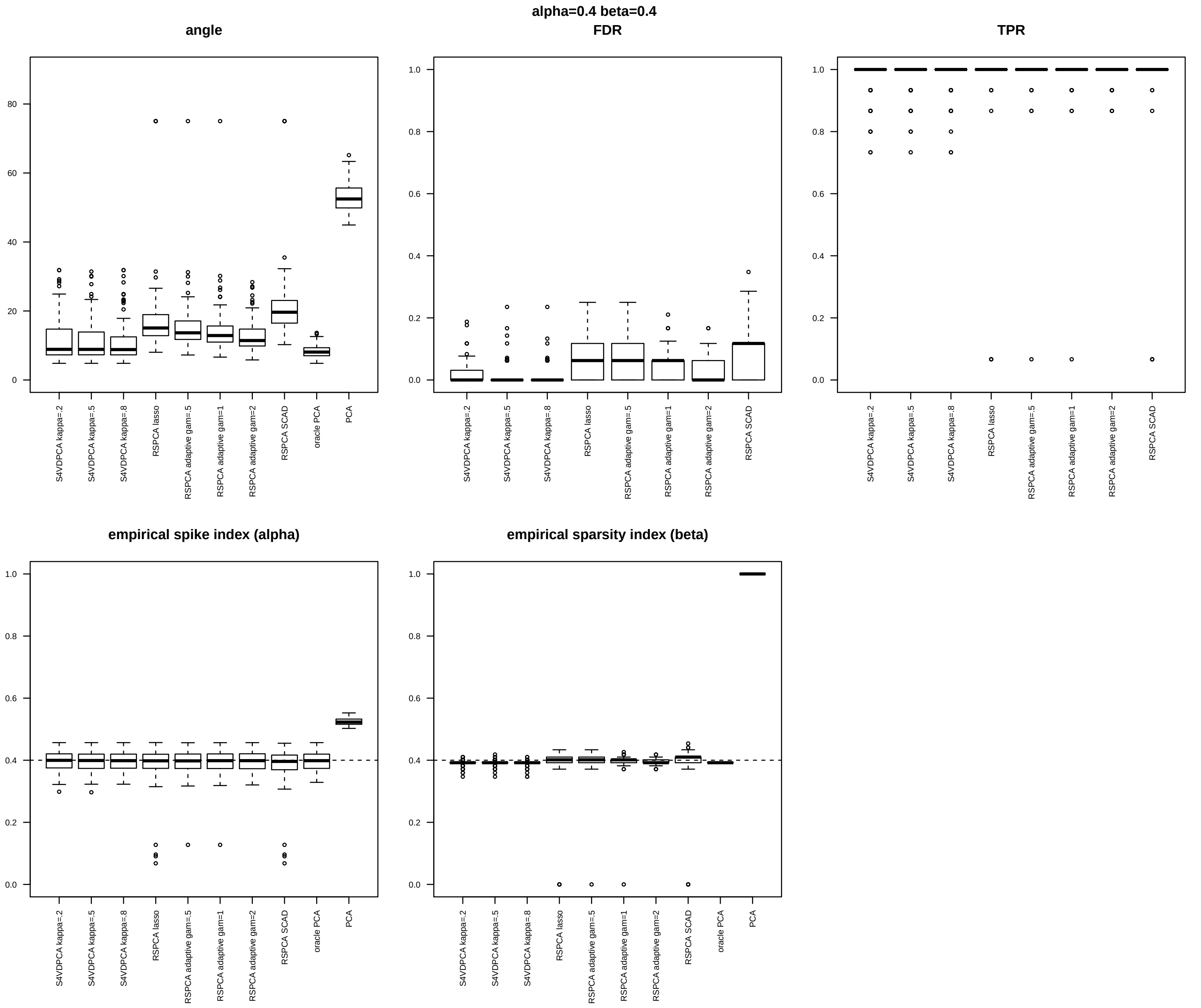

Figure 18: The boxplots display the angle between estimated and true leading eigenvector, FDR, TPR, empirical spike index and empirical sparsity index for 100 simulation runs.

3.4 $\alpha = 0.4$ $\beta = 0.9$

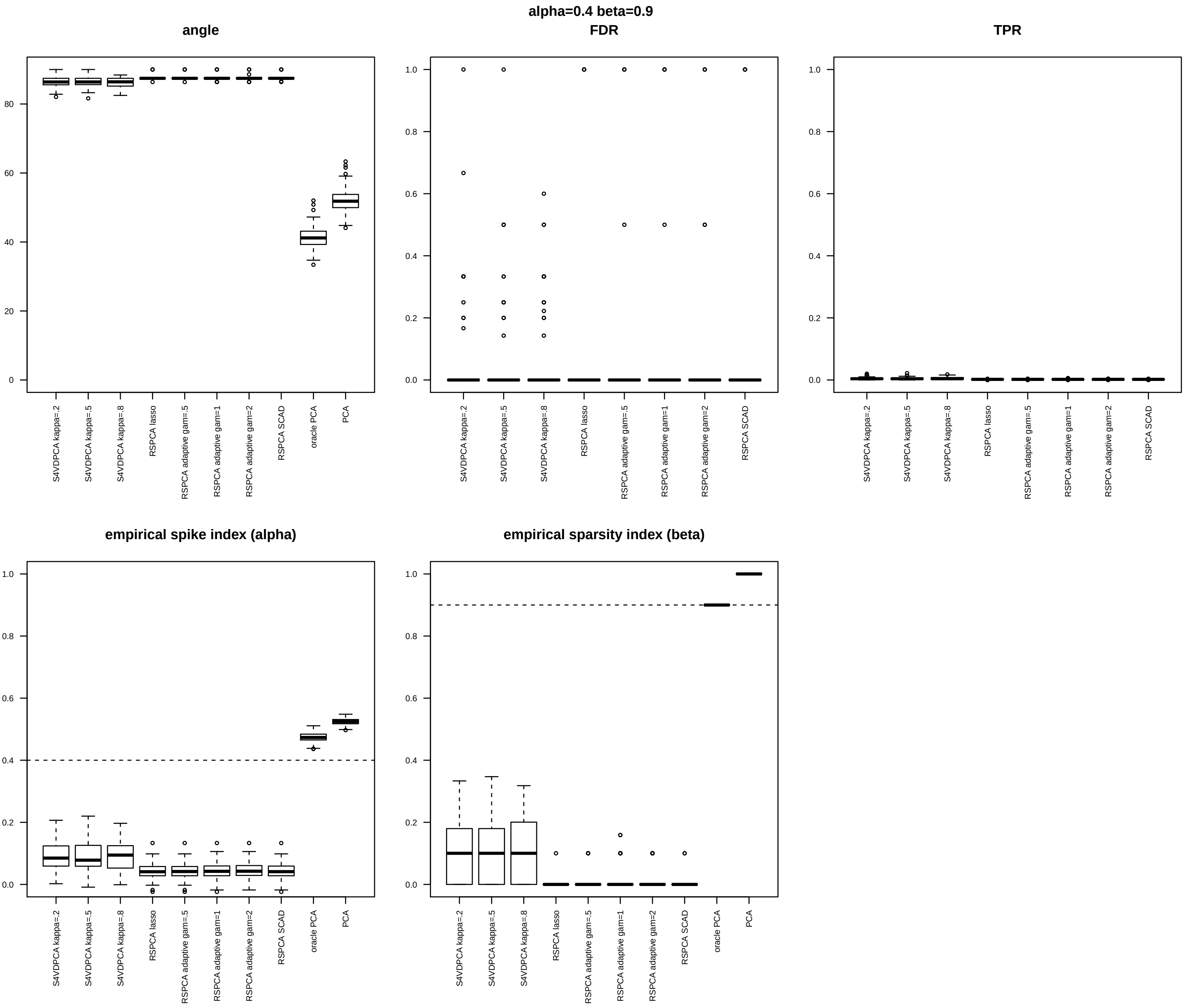

Figure 19: The boxplots display the angle between estimated and true leading eigenvector, FDR, TPR, empirical spike index and empirical sparsity index for 100 simulation runs.

3.5 $\alpha = 0.2$ $\beta = 0.6$

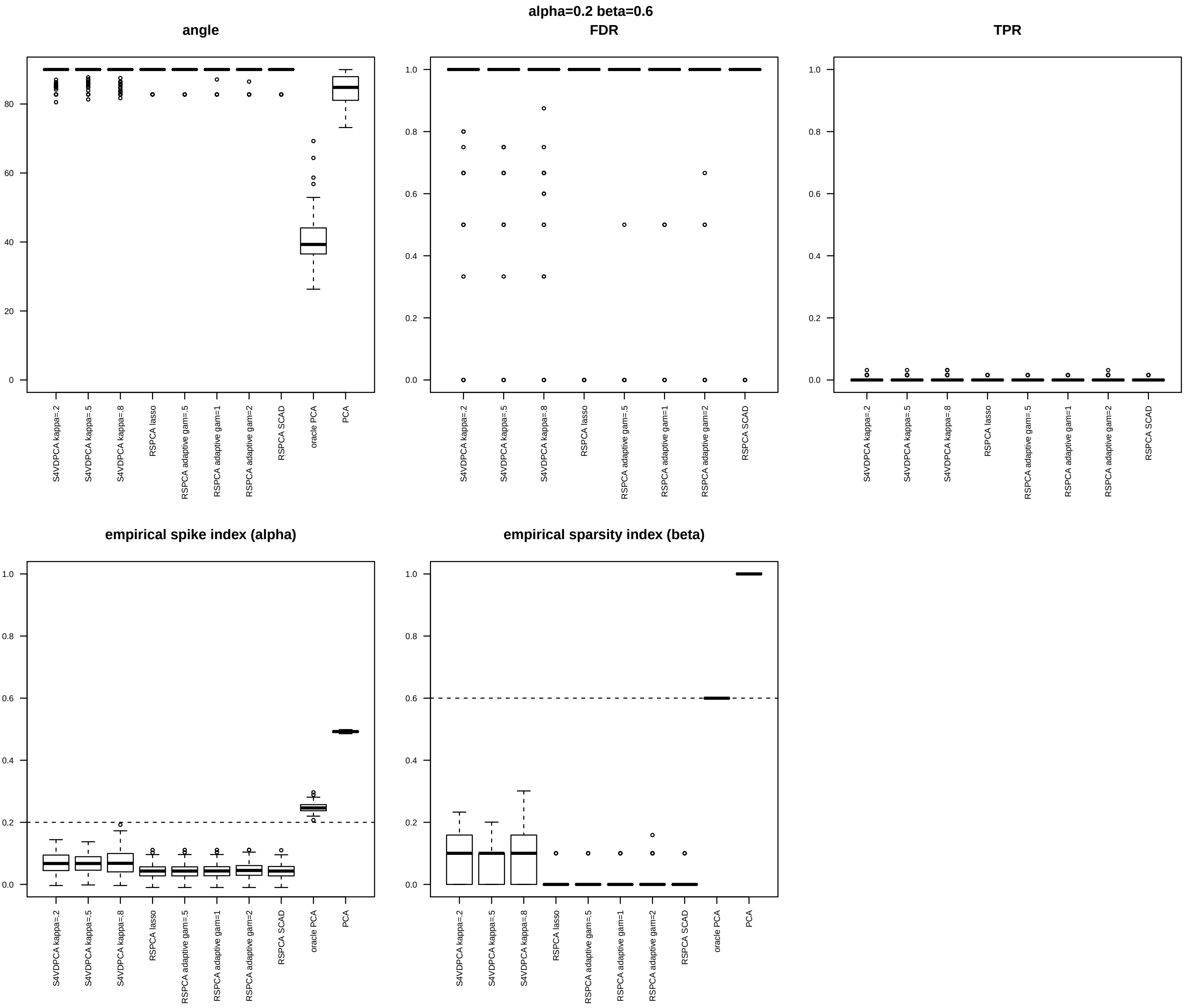

Figure 20: The boxplots display the angle between estimated and true leading eigenvector, FDR, TPR, empirical spike index and empirical sparsity index for 100 simulation runs.

3.6 $\alpha = 0.6$ $\beta = 0.6$

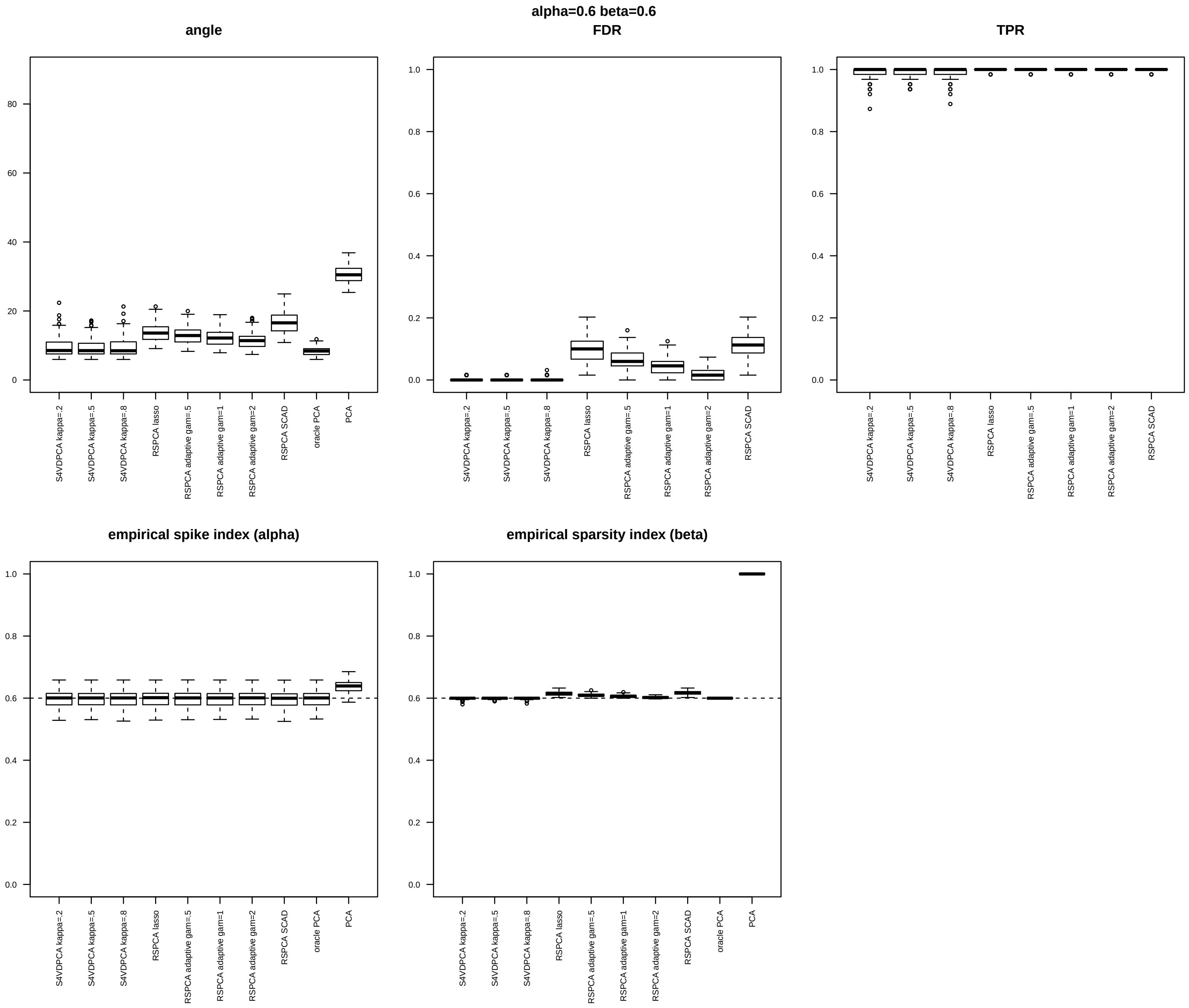

Figure 21: The boxplots display the angle between estimated and true leading eigenvector, FDR, TPR, empirical spike index and empirical sparsity index for 100 simulation runs.

3.7 $\alpha = 0.725$ $\beta = 0.775$

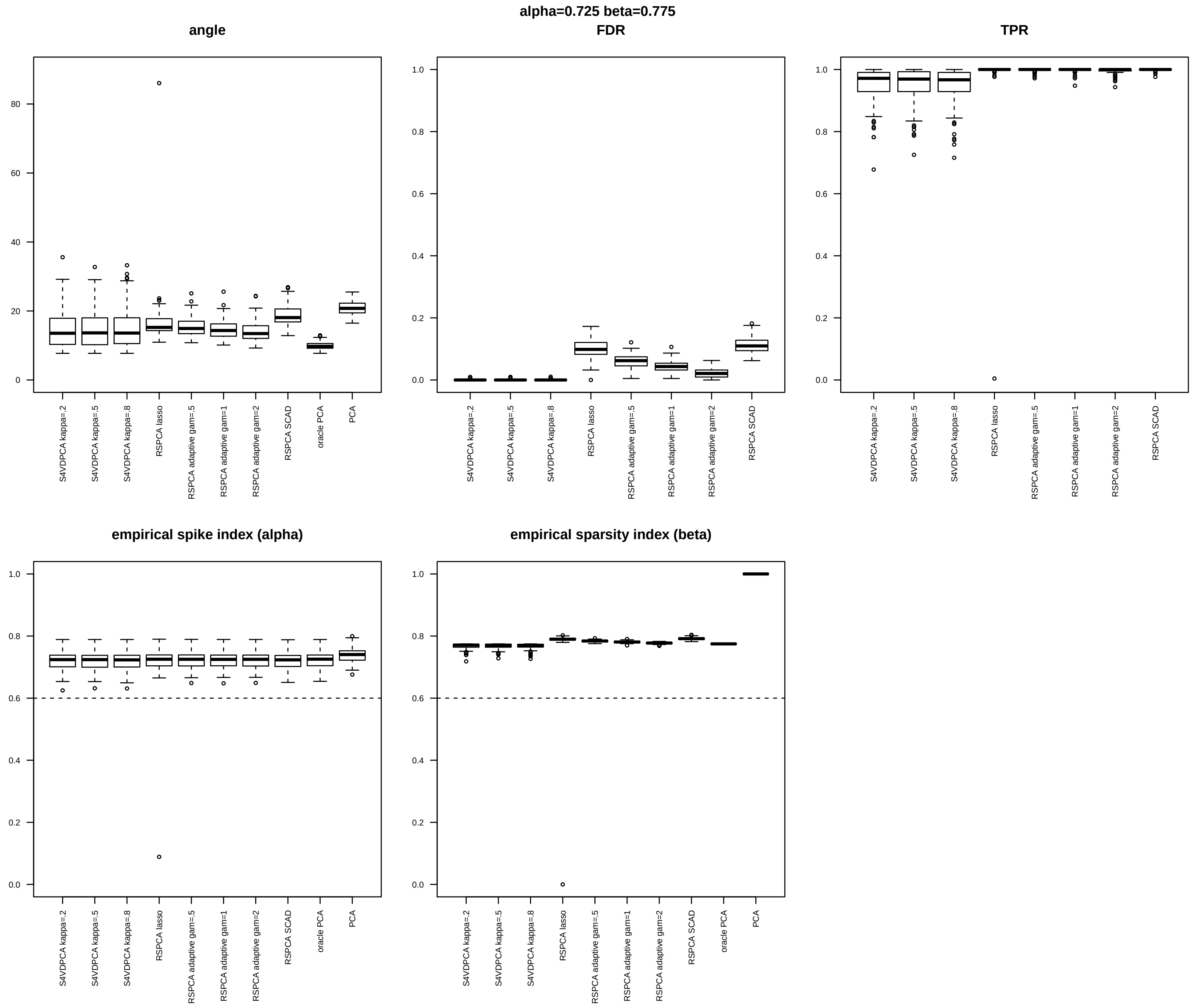

Figure 22: The boxplots display the angle between estimated and true leading eigenvector, FDR, TPR, empirical spike index and empirical sparsity index for 100 simulation runs.

3.8 $\alpha = 0.7$ $\beta = 0.75$

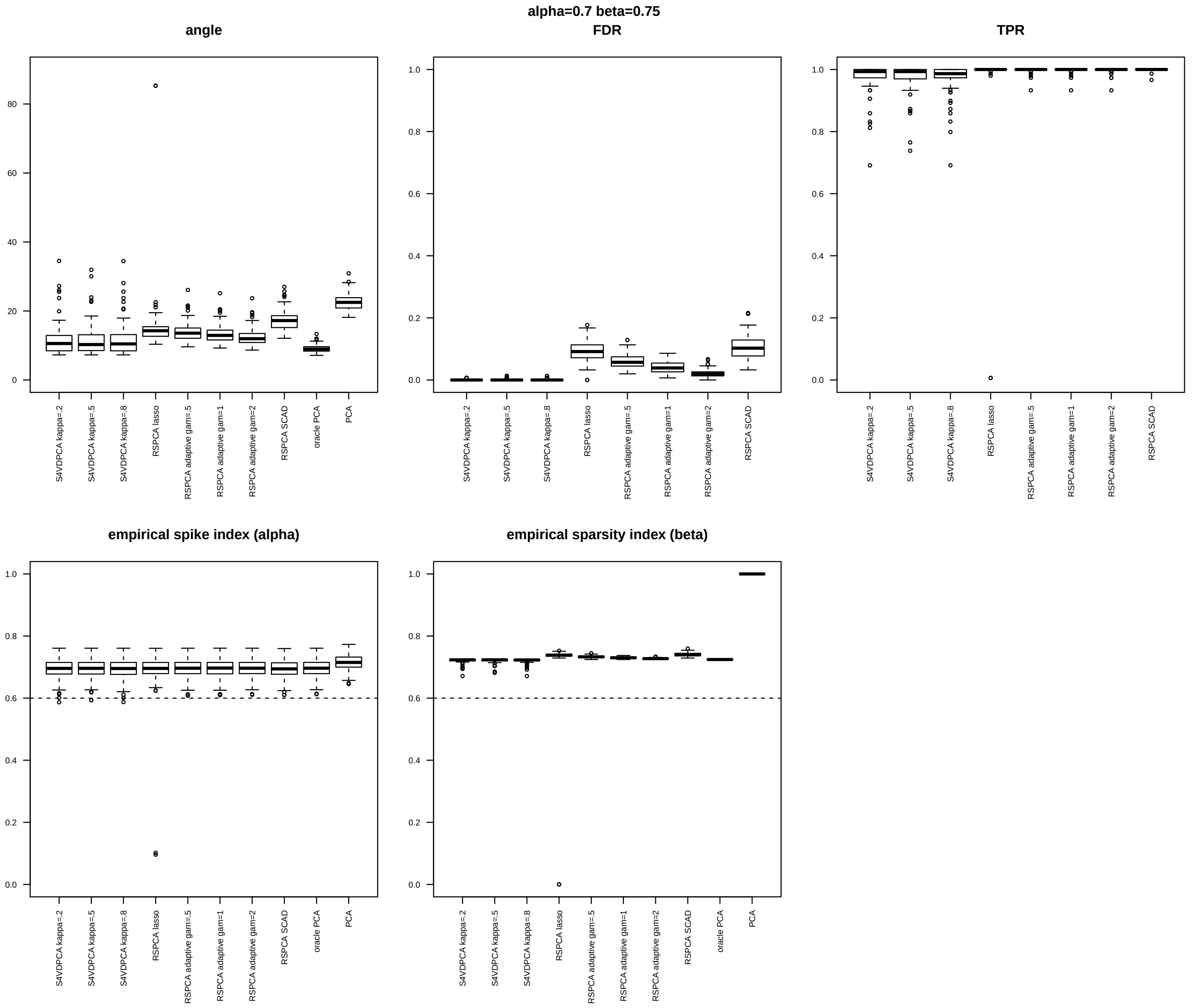

Figure 23: The boxplots display the angle between estimated and true leading eigenvector, FDR, TPR, empirical spike index and empirical sparsity index for 100 simulation runs.
